# Circulating Amyloid and Misfolded Biomarkers in Early Myocardial Injury: A Systematic Review and Epistemic Meta-Analysis

**DOI:** 10.3390/ijms27177956

**Published:** 2026-09-07

**Authors:** Florencio Alejandro Chable-Guerrero, Diana Romero-Zertuche, Cristina Revilla-Monsalve, Lizett Castrejón-Delgado, Nelly F. Altamirano-Bustamante, Myriam Marlenne Altamirano-Bustamante

**Affiliations:** 1 UMAE Hospital de Cardiología del Centro Medico Nacional Siglo XXI, Instituto Mexicano del Seguro Social, Ciudad de Mexico 06720, C.P., Mexico; chable09fag@gmail.com (F.A.C.-G.); dirzcardio@gmail.com (D.R.-Z.); 2 Unidad de Investigación en Enfermedades Metabólicas, UMAE Hospital de Cardiología del Centro Medico Nacional Siglo XXI, Instituto Mexicano del Seguro Social, Ciudad de Mexico 06720, C.P., Mexico; cristina_revilla@hotmail.com; 3 Facultad de Estudios Superiores Zaragoza (FES Zaragoza), Universidad Nacional Autónoma de Mexico (UNAM), Ciudad de Mexico 09230, C.P., Mexico; lizett_castrejon@comunidad.unam.mx; 4 Servicio de Endocrinología, Instituto Nacional de Pediatría, Ciudad de Mexico 04530, C.P., Mexico

**Keywords:** amyloid, serum amyloid type A, hIAPP, death, myocardial infarction, heart failure

## Abstract

Misfolded proteins, including islet amyloid polypeptide (hIAPP), serum amyloid A (SAA), and amyloid-betas (Aβs) such as Aβ1-40 and Aβ1-42 oligomers, collectively referred to in our work as amyloid oligomers, have been implicated in the development of both metabolic and cardiovascular diseases. However, their potential role as early biomarkers of myocardial damage remains insufficiently explored. We conducted a systematic review following PRISMA guidelines and epistemic meta-analysis to investigate the association between misfolded protein oligomers and early myocardial injury. All English- and Spanish-language articles with titles, abstracts, or keywords relevant to the research topic and indexed in at least one of the following databases—PubMed, BIREME, or Web of Science—were included. A comprehensive search across major databases identified 30 eligible studies. Thirty studies met inclusion criteria, in humans and animals, and in vitro. Amyloid oligomers were consistently elevated in patients with acute myocardial infarction, type 2 diabetes, or coronary artery disease compared with controls. In specific individual cohorts, a higher SAA concentration correlated with major adverse cardiovascular events, where it acted as an independent predictor of mortality in reperfused AMI (RR 5.8; 95% CI: 1.3–27.7) and cardiac rupture (OR 8.8; 95% CI: 1.7–25.6). Our findings support the hypothesis that protein misfolding contributes to early myocardial injury and highlight its potential as a novel source of cardiometabolic biomarkers.

## 1. Introduction

Cardiovascular diseases (CVDs) represent the leading cause of mortality worldwide, with an estimated 19.8 million deaths reported by the World Health Organization in 2022 [1]. In Mexico, the magnitude of the problem is also considerable, with 100,710 deaths attributed to CVD in 2024, according to INEGI data [2]. Beyond their health impact, CVDs represent a substantial economic burden: in the European Union, total costs were estimated at €282 billion in 2021, reflecting both direct healthcare expenditures and indirect losses associated with disability and premature mortality [3]. Within this spectrum, acute coronary syndrome (ACS) plays a central role, as it arises from thrombotic occlusion secondary to cholesterol accumulation in the arterial intima and rupture of calcified atherosclerotic plaques. In at-risk populations, early identification is essential and has relied on imaging methods, such as the coronary artery calcium score (CACS), in which higher levels of calcium increase the risk of cardiovascular events, or coronary CT angiography (CCTA), to identify “high-risk” plaque features and quantify the total coronary plaque volume and plaque composition [4]. Biochemical markers also play a role: those related to lipid metabolism, such as low-density lipoprotein (LDL), high-density lipoprotein (HDL), cholesterol remnants, TG/HDL-C ratio, ApoB, and Lp(a)); those linked to low-grade inflammation, such as C-reactive protein and interleukin-6 (IL-6); and finally, those that reflect early renal impairment, including the glomerular filtration rate and microalbuminuria. Taken together, these parameters provide an integral clinical and prognostic overview, contributing to risk stratification and supporting the implementation of more timely preventive and personalized interventions.

Ischemic heart disease is the leading cause of death worldwide and the primary contributor to heart failure [5,6]. Patients with ischemic heart disease and heart failure often share common risk factors; many of these risks fall under the umbrella of metabolic syndrome, which includes conditions such as insulin resistance, obesity, and hypercholesterolemia [7]. These conditions, regardless of cardiac contractile function, contribute to myocardial damage through chronic inflammation [8]. This inflammatory cascade is well characterized in the context of atherosclerosis; the process begins with endothelial injury and LDL accumulation, followed by immune cell recruitment, extracellular matrix (ECM) deposition, and the release of inflammatory mediators, which together compromise vascular integrity and repair mechanisms [9]. Recognizing atherosclerosis as an inflammatory disease has shifted attention toward identifying biomarkers that could not only predict cardiovascular outcomes but also reflect the presence and progression of disease [10,11].

More recently, misfolded proteins, particularly those with amyloidogenic properties, have emerged as contributors to these chronic disease states and are called conformational diseases. Within this framework, the evaluation of circulating toxic aggregates encompasses a broad spectrum of misfolded proteins and specific molecular subtypes, including human islet amyloid polypeptide (hIAPP)—specifically evaluated through its naturally occurring circulating amyloid oligomers, designated as real human islet amyloid polypeptide amyloid oligomers (RIAOs)—as well as other amyloid aggregates like serum amyloid A (SAA) aggregates, and amyloid-beta (Aβ1-40 and Aβ1-42) oligomers. Although considerable attention has focused on proteins like hIAPP in the context of T2D and neurodegenerative diseases, evidence is mounting that these and other misfolded proteins may play active roles in cardiovascular injury [12]. An intriguing and underexplored phenomenon is the ability of these misfolded proteins to circulate systemically, damaging distant tissues beyond their site of origin. For example, oligomers of human islet amyloid polypeptide (hIAPP) produced in the pancreas—also known as RIAOs—circulate through the bloodstream and serve as early biomarkers of β-cell failure, while also exerting harmful effects on distant tissues such as myocardial cells [10]. RIAOs can be isolated, characterized, and quantified using cost-effective immunoassays. Importantly, RIAOs do not act in isolation; emerging evidence suggests that they are capable of co-aggregating with other misfolded proteins, giving rise to hybrid amyloid complexes in a phenomenon known as amyloid cross-seeding. However, this intricate process of co-aggregation remains poorly understood. As such, it presents a critical frontier in proteomic research and poses a significant challenge for the classification of complex systemic disorders—including cardiovascular and neurodegenerative diseases [13,14].

Despite increasing research interest, the precise aggregation–oligomerization–fibrillization process by which circulating amyloidogenic proteins contribute to myocardial damage, co-aggregate with other proteins, and disrupt systemic homeostasis remains unclear [15]. This review aims to explore the evidence of protein misfolding in patients with acute myocardial infarction, focusing on how these proteins—originating from metabolically active or inflamed organs such as the pancreas or adipose tissue—may target the heart. Thus, the guiding research question is: “Can non-native (aberrant) folding forms of amyloid oligomers protein be detected in greater abundance in patients with myocardial dysfunction?”. Additionally, this systematic review explores the novel concept of the heart as a “target organ” in protein conformational diseases (PCDs), with implications for biomarker discovery and cardiovascular risk stratification [16]. The central objective of this research is to determine whether the accumulation of amyloid oligomers in cardiac cells is greater in patients with myocardial injury. In doing so, this study aims to bridge fundamental research on protein misfolding with clinical applications, accelerating the identification of early biomarkers of myocardial cell failure. CVDs like other PCDs are catastrophic—not only due to their clinical severity but also because of the immense economic and social burden they place on families, healthcare systems, and governments. Advancing our understanding of molecular events such as amyloid aggregation in cardiac tissue may open new avenues for early diagnosis, risk stratification, and therapeutic intervention in PCD.

## 2. Results

**Figure 1:**
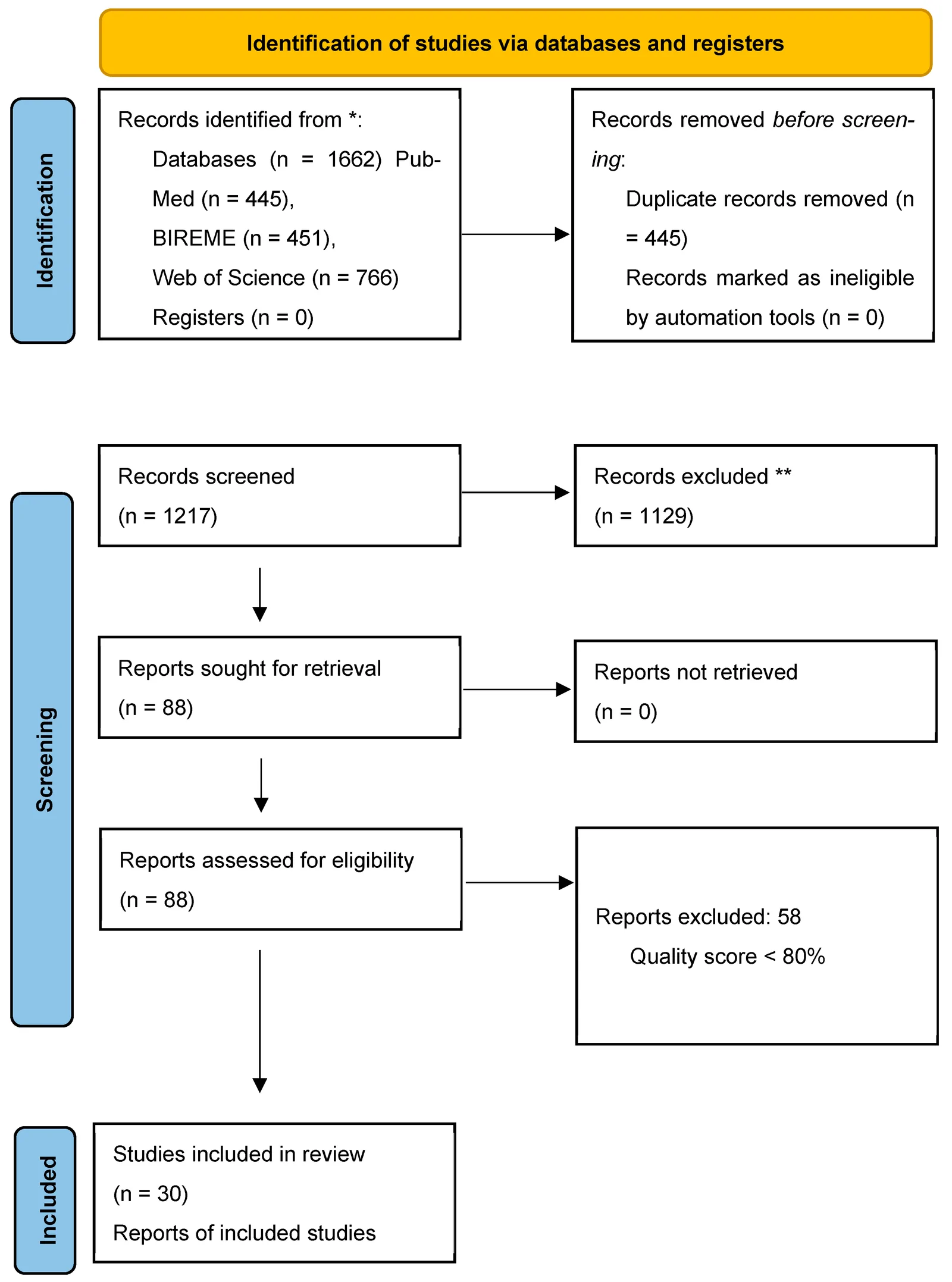
PRISMA flow diagram of study selection. Preferred Reporting Items for Systematic Reviews and Meta-Analyses (PRISMA) flowchart showing the identification, screening, eligibility assessment, and inclusion of studies. The diagram summarizes the search process across multiple databases and reasons for exclusion at each stage.

A total of 1662 articles were initially identified from three electronic databases. The identification, screening, eligibility assessment, and final inclusion of these studies followed the Preferred Reporting Items for Systematic Reviews and Meta-Analyses (PRISMA) guidelines (Figure 1).

**Figure 2:**
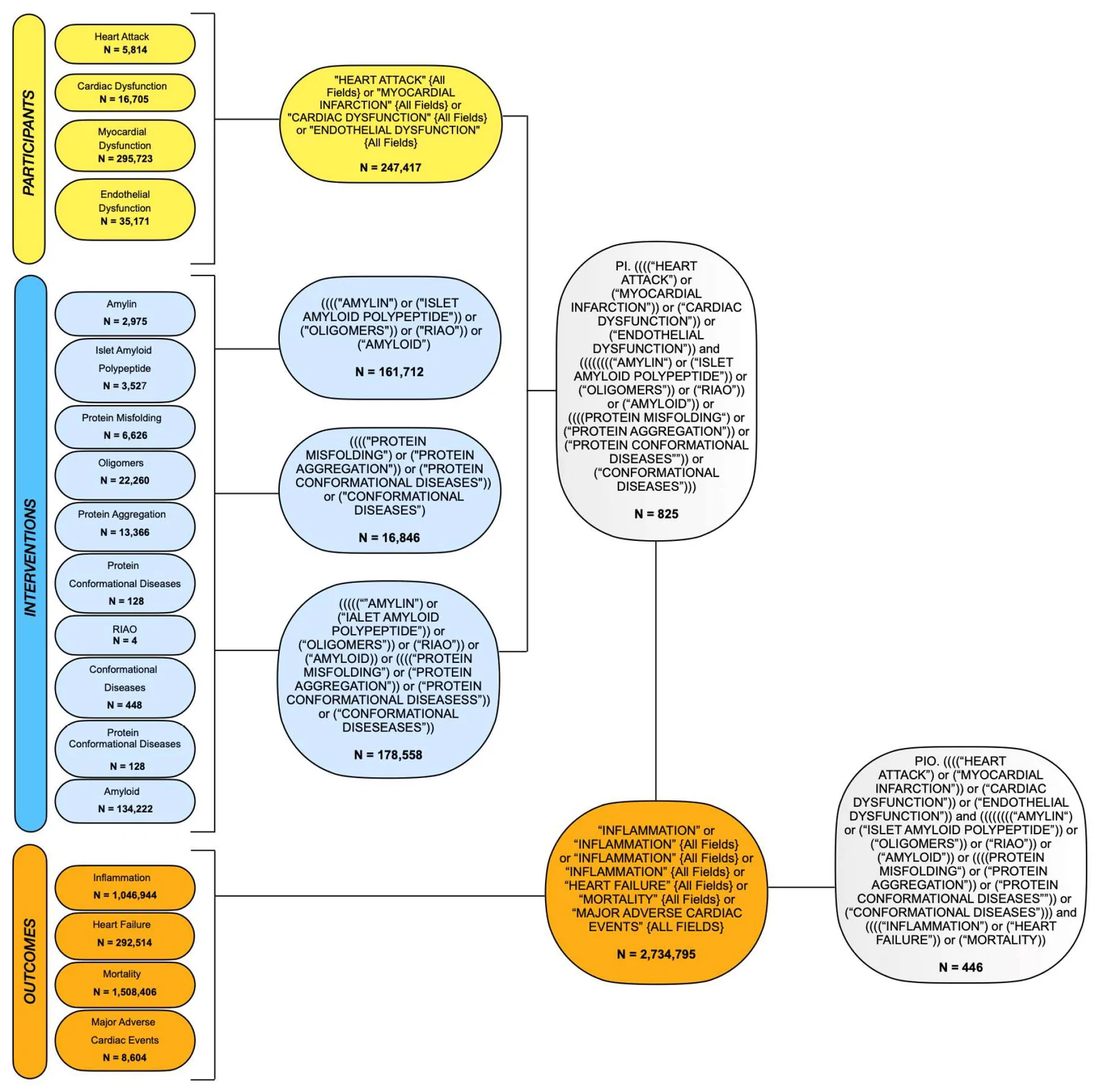
Flow diagram of the selection process. A representative decision diagram illustrating the selection of articles retrieved from the PubMed database using the PIO strategy.

To select the most relevant evidence, we conducted a four-stage screening process based on our research question, detailing the retrieval strategy across databases using the PIO framework (Figure 2).

**Figure 3:**
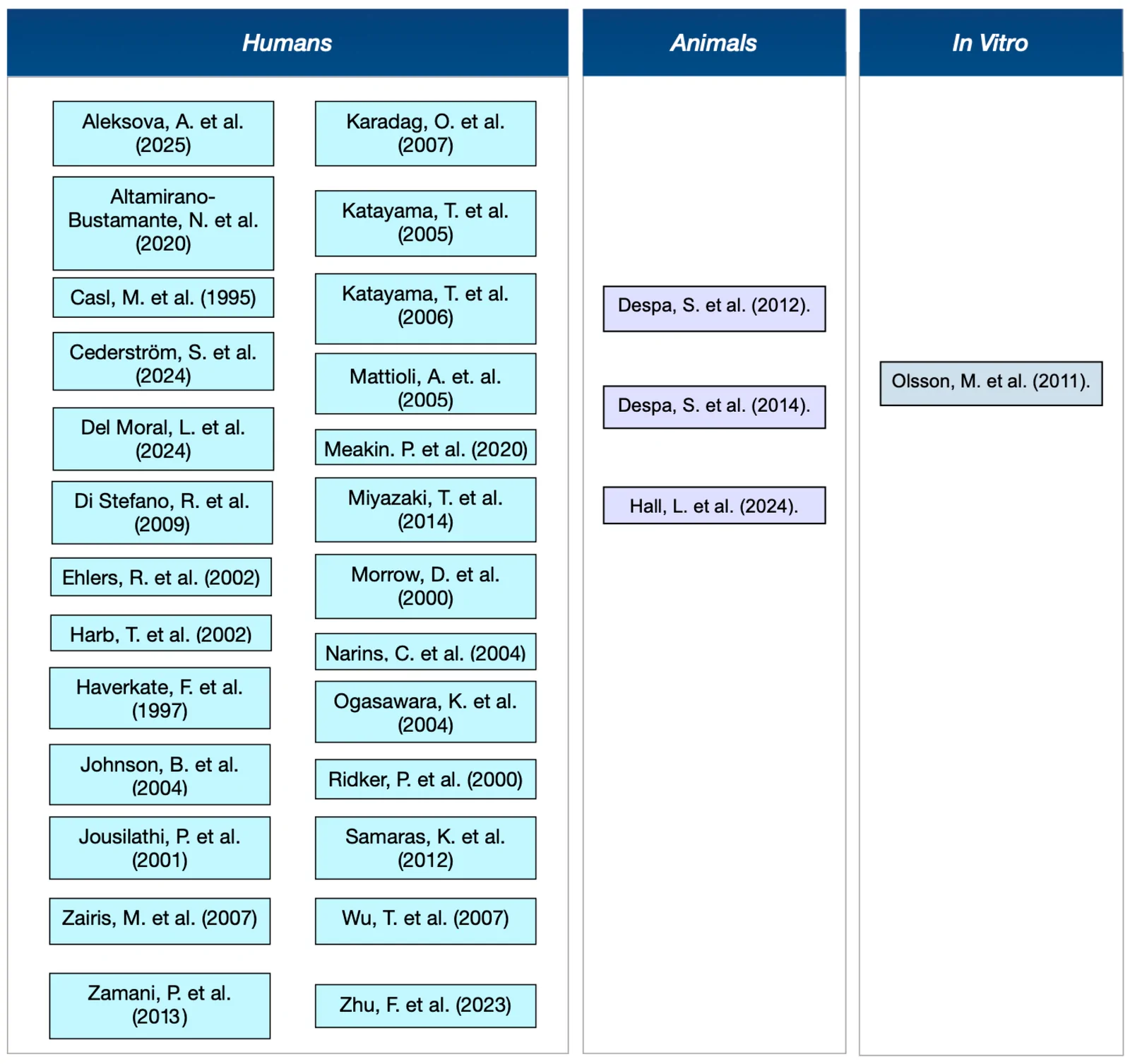
Distribution of studies according to design. Characterization of the 30 studies included in the systematic review and epistemic meta-analysis, categorized according to study design (e.g., humans, animals, in vitro) [16,17,18,19,20,21,22,23,24,25,26,27,28,29,30,31,32,33,34,35,36,37,38,39,40,41,42,43,44,45].

First, duplicate articles were removed. Next, articles irrelevant to our topic were excluded, and 4 articles were added manually, leaving a final total of 30 relevant studies categorized according to their design—comprising human cohorts, animal models, and in vitro assays (Figure 3).

**Figure 4:**
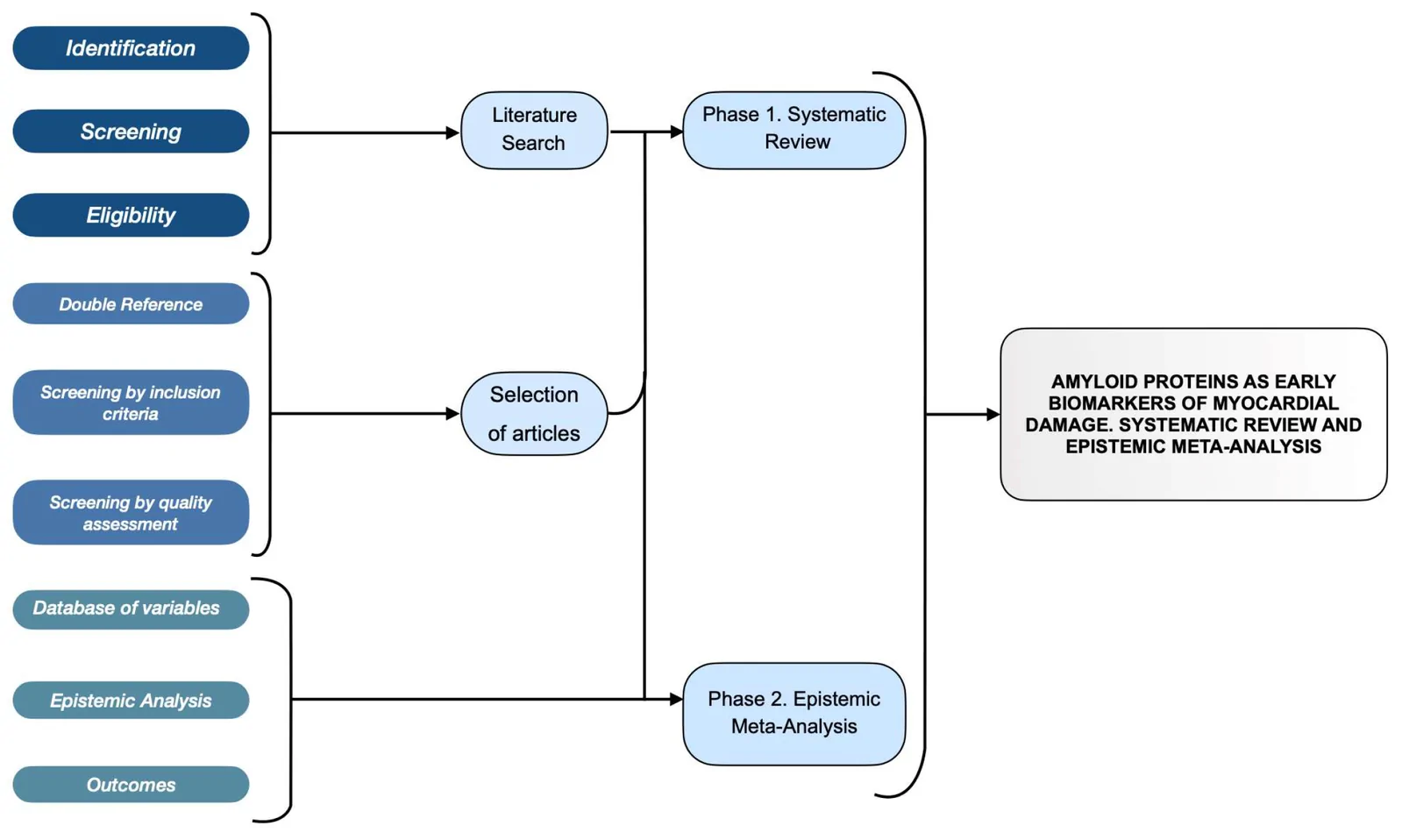
Flow chart of the systematic review and epistemic meta-analysis. Schematic representation of the process used for the systematic review and epistemic meta-analysis, including initial database searches, study screening, full-text assessment, and final inclusion.

The overall methodological workflow and schematic process of the systematic review and epistemic meta-analysis are outlined in Figure 4.

### 2.1. Risk of Bias in Studies

Regarding the quality of the included studies, we considered the heterogeneity of the analytical methods used to quantify oligomers across studies. These included the sandwich ELISA method for measuring Aβ1-40 levels, micro-ELISA, microparticle enzyme immunoassays for determining serum amyloid A (SAA) levels, particle-enhanced immunonephelometry for SAA measurement, and high-sensitivity ELISA kits for quantification of Aβ-40 and Aβ-42. With respect to risk of bias, none of the included studies were randomized clinical trials or quasi-experimental designs. Among the human studies included, the most common design was cohort studies (56.7%), followed by cross-sectional studies (23.3%), case–control studies (10%), and experimental studies (10%).

Quality appraisal instruments are described in Section 4.2, per-study scores can be found in Table S3A–C.

The quality of cross-sectional studies ranged from low to regular, corresponding to a high to moderate risk of bias. In contrast, most cohort and case–control studies were of great quality and therefore had a low risk of bias. In animal model studies, unclear risk of bias predominated across most of the domains assessed.

### 2.2. Integral Epistemic Meta-Analysis Algorithm

**Figure 5:**
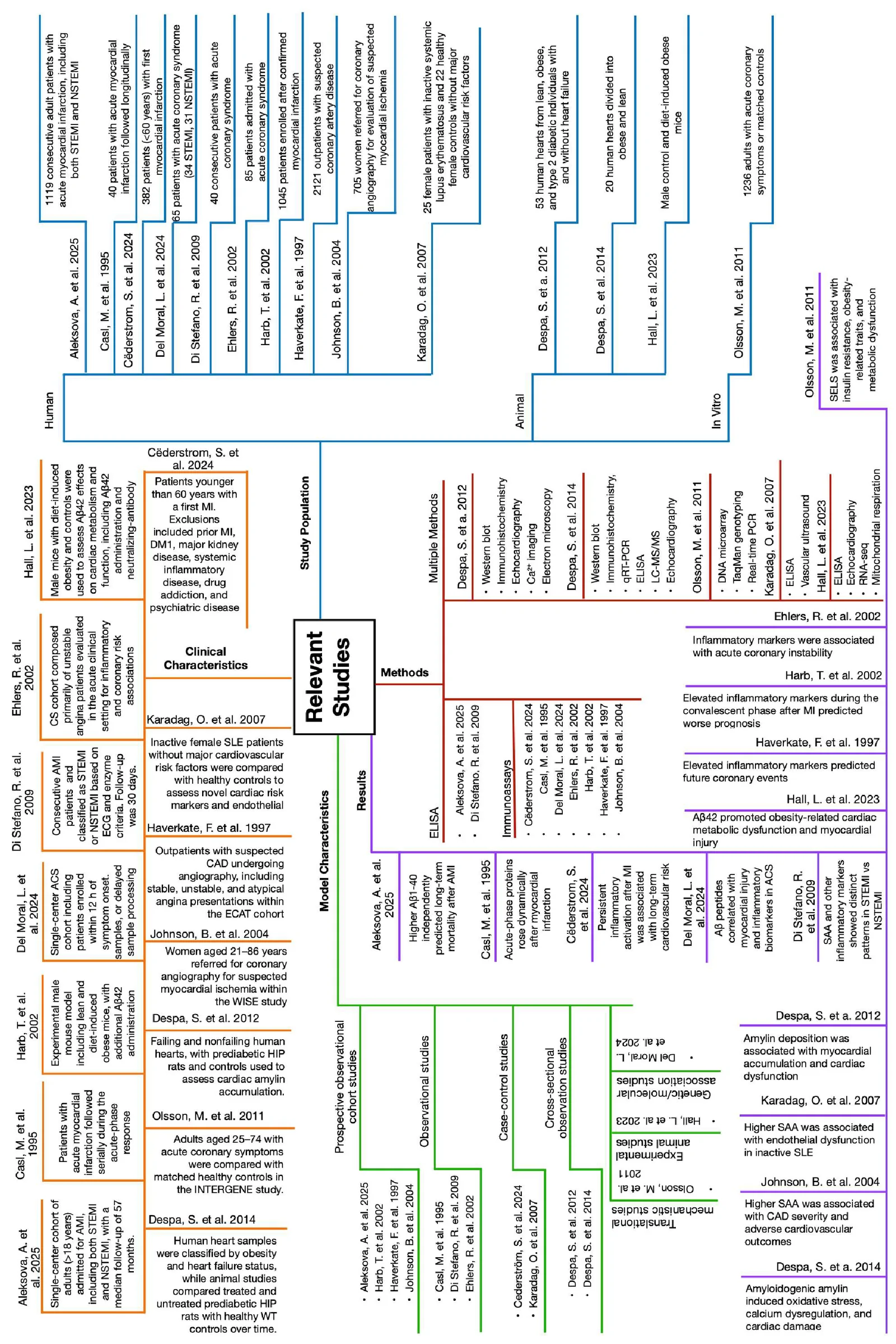
This mind map summarizes the most relevant information from the representative studies, including 10 human studies, 3 animal studies, and 1 in vitro study. It is designed to condense and integrate the key findings, methodologies, and experimental approaches of each article, highlighting their most important contributions in a concise and structured manner.

The 30 studies selected through the systematic review were analyzed following an integral epistemic meta-analysis algorithm encompassing five steps. First, the studies were classified by population type—human, animal, or in vitro—and by the primary oligomer reported, with particular attention placed on studies reporting on SAA and Aβ1-40. Second, all relevant data were systematically extracted from each article and registered in a structured spreadsheet database, which constituted the primary instrument of the analysis. The database was organized with one row per article and columns grouped into five clearly defined categories: general study information (study identifier, journal, and year of publication); population characteristics (sample size, group subdivisions, number of participants per group, and patient age with its corresponding uncertainty measure per group); oligomer data (principal oligomer or protein analyzed, and whether SAA or Aβ1-40 were specifically reported); comorbidities (number of mortality events and prevalence of conditions including diabetes mellitus, heart failure, and myocardial infarction); and reported outcomes (main results and associations described by the study). Third, to complement the database and provide an integrated visual representation of the evidence, mind maps were constructed for all included studies following a common baseline-scaffolding template, as described in the following section. Fourth, the studies were analyzed by a mixed-method approach—comparing all articles across specific aspects simultaneously—and by subgroup according to population type and oligomer of interest. Fifth, the synthesis of the findings was used to address the research question regarding the potential of amyloid oligomers as early biomarkers of myocardial damage and their variation across patients with and without diabetes mellitus; see Figure 5.

### 2.3. Mind Maps of the Epistemic Meta-Analysis

**Figure 6:**
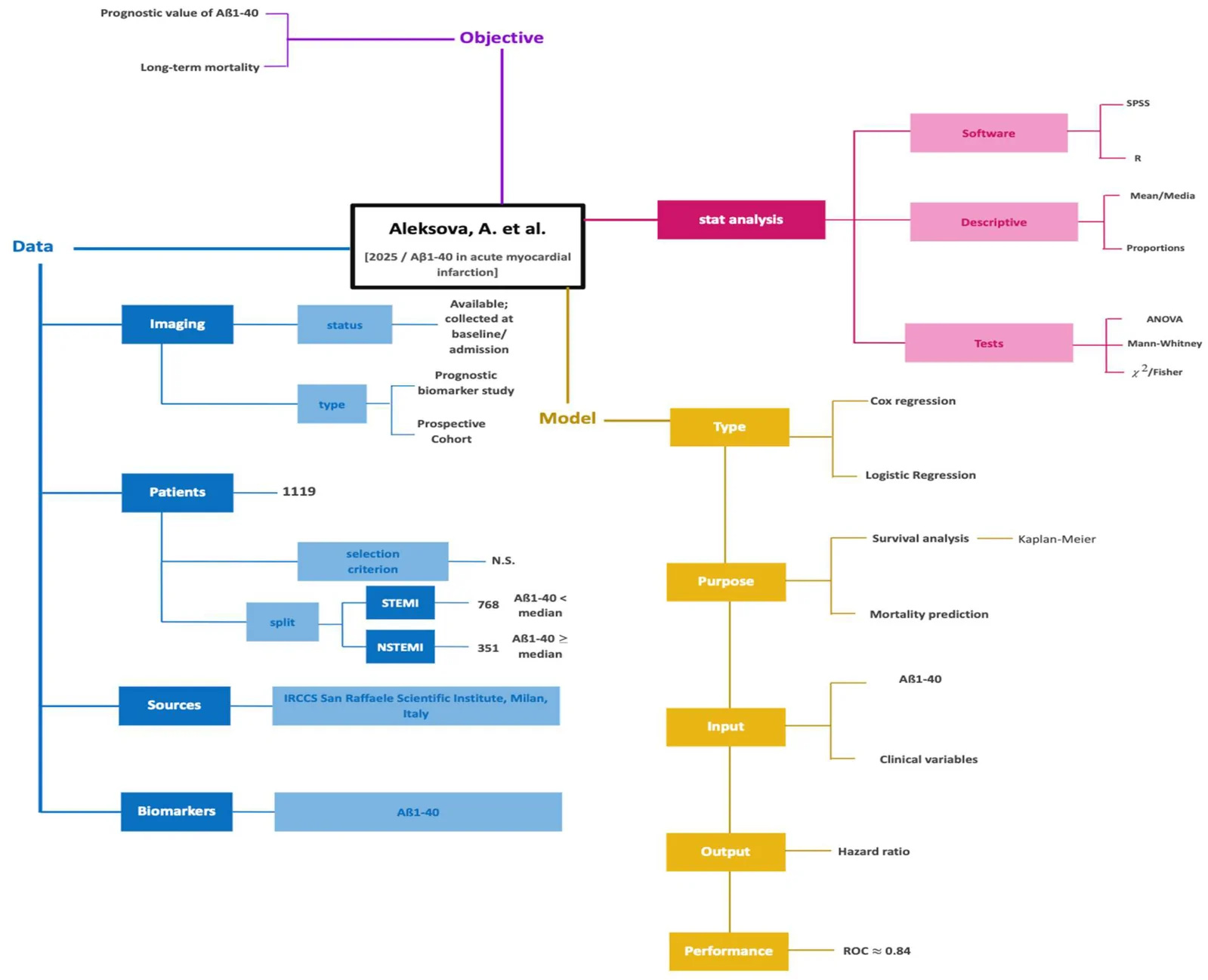
This mind map presents a prospective human cohort study investigating the prognostic value of Aβ1-40 as a biomarker in patients with acute myocardial infarction. It summarizes the study design, including patient population (STEMI and NSTEMI), data collection at admission, and biomarker quantification. The analytical framework, based on survival and regression models, is outlined alongside statistical methods and performance metrics (e.g., ROC analysis). The map emphasizes the study objective—evaluating Aβ1-40 as a predictor of long-term mortality—and provides a concise synthesis of how clinical variables and biomarker levels were integrated to assess prognostic outcomes [46].

**Figure 7:**
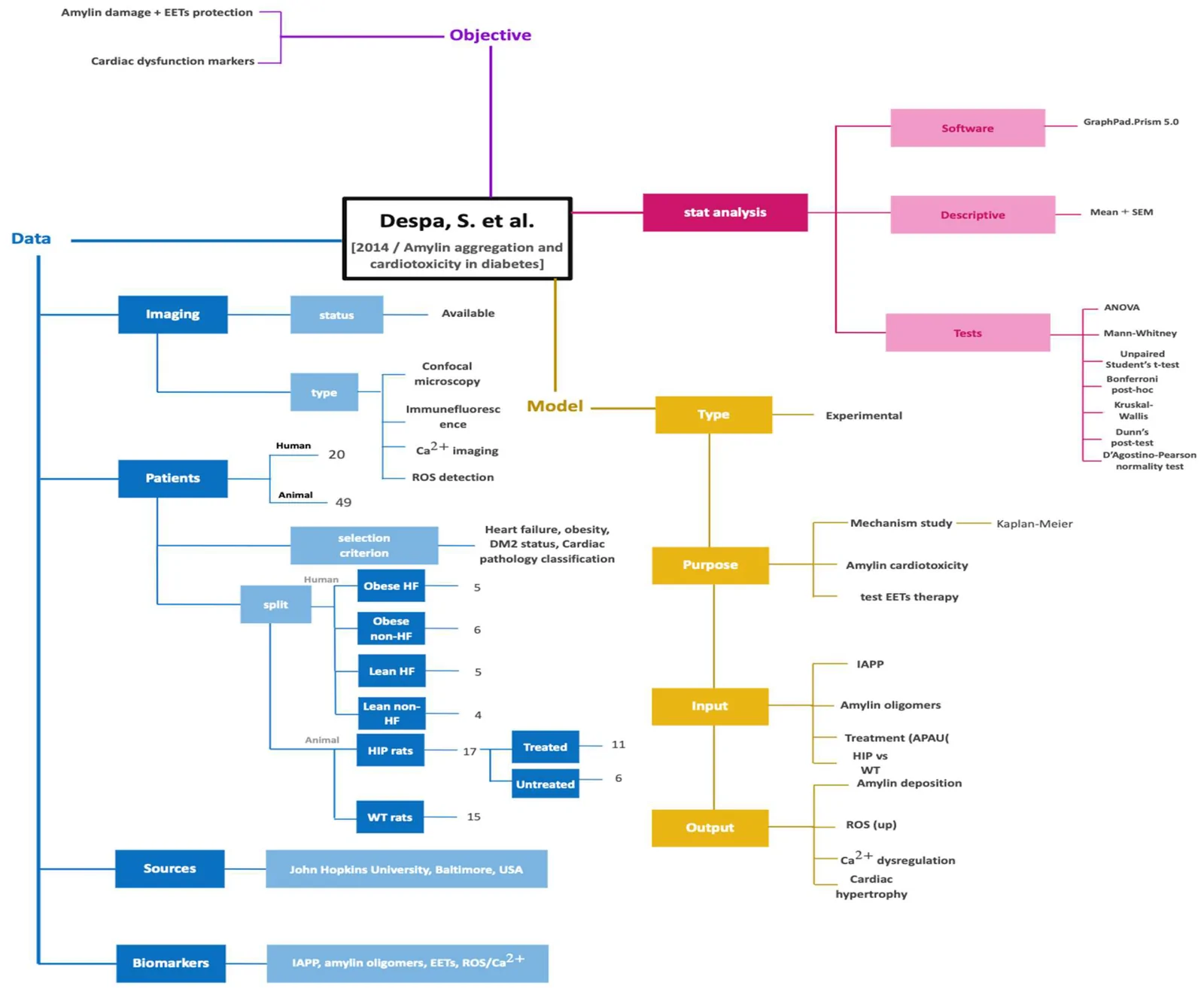
This mind map summarizes an experimental study combining animal models and human tissue analysis to investigate amylin aggregation and its role in cardiotoxicity. It outlines the experimental design, including animal models (HIP and wild-type rats), human samples, and treatment conditions, as well as imaging techniques such as confocal microscopy and Ca^2+^ imaging. The map highlights the study objective—understanding amylin-induced cardiac damage and evaluating epoxyeicosatrienoic acid-based protective mechanisms—and details the analytical methods and outputs, including oxidative stress, calcium dysregulation, and cardiac hypertrophy. Overall, it provides a concise overview of the mechanistic insights into amylin-related cardiac dysfunction [28].

**Figure 8:**
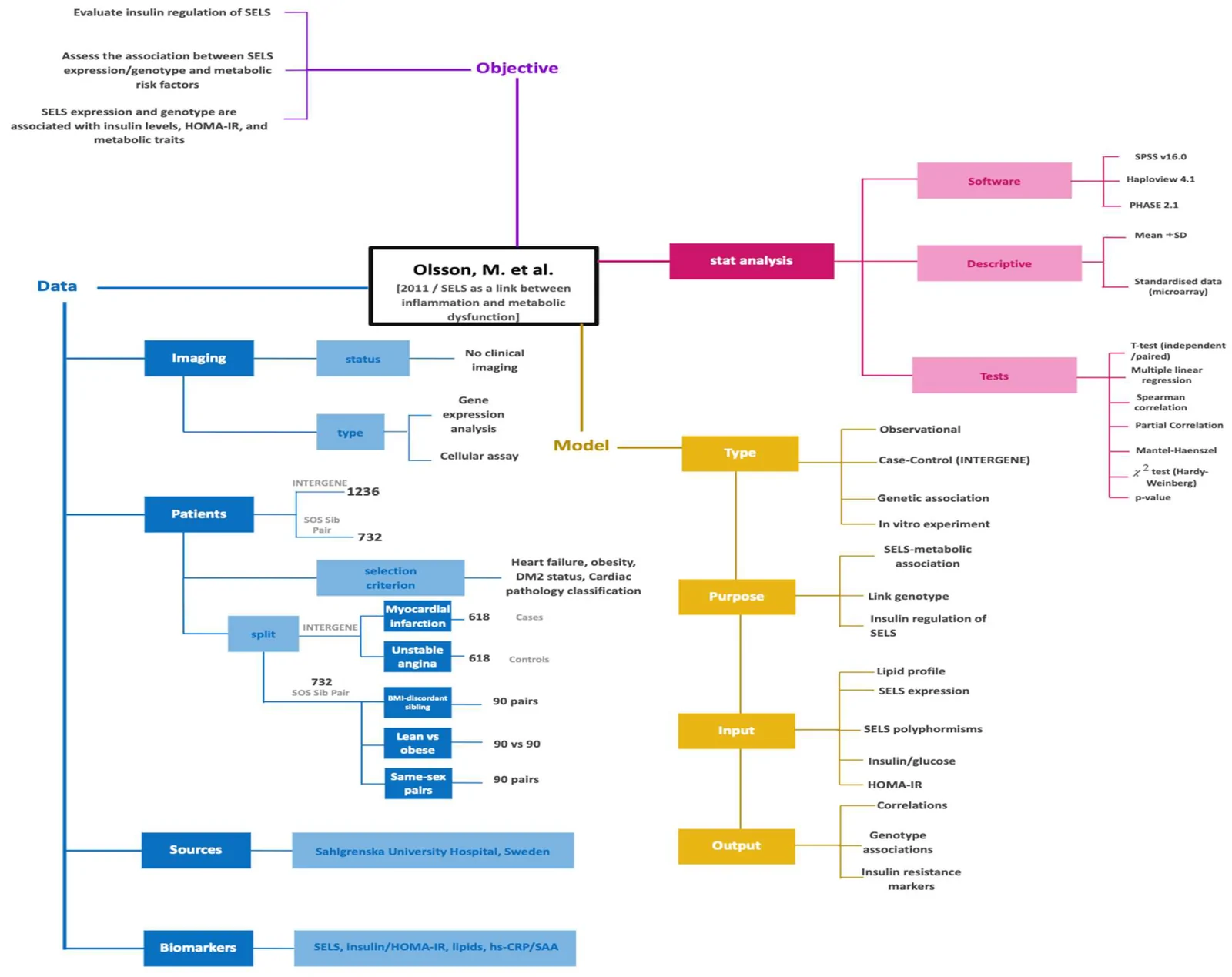
This mind map summarizes an in vitro experimental component evaluating the regulation of selenoprotein S expression in human adipocytes. It integrates key elements of the study, including the use of isolated adipocytes, insulin stimulation conditions, and gene expression analysis methods (microarray and qPCR). The map highlights the objective of assessing the effect of insulin on SELS expression, along with the statistical approaches used to evaluate changes in expression levels. Overall, it provides a concise and structured overview of the experimental design and demonstrates the role of insulin in modulating SELS expression at the cellular level.

The mind maps served as a visual and hierarchical complement to the spreadsheet database, enabling an integrated representation of each study’s key elements within a common structural framework. A total of four mind maps were constructed: the first mind map provided a panoramic view of the most relevant studies in the corpus, encompassing the ten most relevant articles from the human population group, the three animal model studies, and the one in vitro study (Figure 6, Figure 7 and Figure 8).

In this overview map, each article was represented in a condensed format, capturing its essential characteristics—population type, oligomer of interest, main clinical or experimental outcome, and key findings—so that the constant and variable components of the evidence base could be identified at a glance and the studies compared across population types within a single visual structure. The remaining three mind maps each corresponded to a single representative article: one selected from the human population studies, one from the animal model studies, and one from the in vitro study, and were constructed with a greater level of detail than the overview map. These detailed mind maps followed the same baseline-scaffolding template as the overview but expanded each branch to capture the full methodological and results information of the corresponding article, including study design, group subdivisions, measurement approach, oligomer levels reported, and associated clinical or experimental outcomes.

Although human studies constitute the majority of the corpus, animal and in vitro studies were included to provide basic research mechanism evidence underlying the observed associations in human cardiovascular disease.

The mind map structure used in this study was adapted from the baseline template used in Chilaca-Rosas et al. [23]. It organizes information around two central axes: what was done—encompassing study characteristics, population, oligomers measured, and clinical or experimental outcomes—and how it was done—encompassing study design, group subdivisions, and measurement approach. Given that the present work focuses on the characterization of amyloid oligomers as biomarkers rather than on the development of artificial intelligence models, the branches pertaining to machine learning algorithms and model performance were replaced by branches corresponding to oligomer quantification methods, reported biomarker levels, and clinical associations. This structural adaptation preserved the analytical logic of the original template while tailoring it to the specific objectives of the present study.

### 2.4. Role of Amyloid Proteins in Cardiovascular Disease

For a comprehensive understanding of the research landscape, Table S1 summarizes the research characteristics of all 30 studies (Figure 3). The largest study, which was conducted by Ridker [16], included 28,263 postmenopausal women without a prior history of cardiovascular disease and focused on identifying new biomarkers associated with cardiovascular risk. The smallest study, performed by Kiernan [24], analyzed six patients, examining the relationship between inflammation and cardiovascular disease markers. Most studies (76.7%) investigated SAA, amyloid beta (Aβ) peptides such as Aβ40 and Aβ42 (13.3%), and hIAPP or amylin (10%) [17,25]. For measuring oligomers, ELISA was the most frequently used method (43.3%), followed by immunonephelometry (33.3%), automated microparticle enzyme immunoassays and general clinical assays (13.3%), Western blot and immunohistochemistry (6.7%), and microarrays (3.4%). It is noteworthy that misfolded proteins can aggregate, forming toxic oligomers and amyloid fibrils inside and outside cells. These aggregation processes contribute to various diseases, including cancer and cardiovascular diseases.

### 2.5. Translational Medicine in Cardiovascular Disease: Bridging Molecular Biology and Clinical Practice

The history of amyloid oligomers in cardiovascular disease has been progressively constructed. The possibility that aberrant forms of protein folding can be detected in greater abundance in patients with myocardial dysfunction has been the subject of growing interest in translational research, given that their involvement in different pathophysiological pathways can lead these proteins to their transition from molecular biology to their applicability in clinical practice. Amyloid oligomers derived from proteins such as hIAPP, Aβ, and SAA represent highly toxic non-native conformations with properties similar to those of prions and with a strong tendency to aggregate in tissues; this is because, unlike mature fibrils, they are more soluble and have greater capacity to interact with cell membranes, altering intracellular calcium handling and promoting apoptosis and myocardial remodeling [26]. Experimental data from animal and in vitro studies have provided the first evidence of this phenomenon. Studies published since 2011 have highlighted the accumulation of these proteins at the cardiovascular level as a preceding factor of cardiomyocyte dysfunction, with clinical translation in its different varieties, whether at the level of epicardial arteries as a destabilizer of atherosclerotic plaque and ischemic heart disease, inflammation, and pro-arrhythmogenic factor, or as diastolic or systolic myocardial dysfunction, with its translation into heart failure of different degrees. Initially, Olsson et al. contributed to this narrative by showing that the SAA protein can interact with selenoprotein S (SELS), which genetically links protein accumulation with inflammation, insulin resistance, and cardiovascular risk [26,27] (Figure 6). A study conducted by Despa et al. in transgenic rats for human amylin demonstrated that hyperamylinemia promotes the formation of abnormal oligomers of different sizes—12, 16, 32, and up to 64 kDa—that accumulate in the sarcolemma and alter cardiac function early, even before the manifestation of hyperglycemia, that is, in the prediabetic phase. This finding suggests that the detection of these aberrant conformations is possible and precedes the appearance of endothelial or clinical cardiovascular dysfunction As confirmed by Despa et al. with an analysis of 53 human hearts amylin oligomers were present in 100% of the failed hearts of patients with T2D (n = 25) and in those of obese patients who developed diabetes in the year after transplantation (n = 8). Even in non-failed hearts of overweight individuals (n = 8), small oligomers were identified, suggesting an initial state of accumulation. Moreover, the finding that higher-molecular-weight forms (32–64 kDa) were detected exclusively in the hearts of patients with diabetic heart failure provides key evidence to understand disease progression [26,28] (Figure 7). This pattern not only indicates that amyloid oligomers increase in number, but also that they evolve into structurally more complex and potentially more toxic conformations as clinical deterioration advances. The transition from small oligomers, observed in non-failed hearts of overweight individuals, to high-molecular-weight aggregates in diabetic heart failure suggests a pathological gradient that accompanies the clinical course of myocardial dysfunction. Such a gradient is particularly revealing because it establishes a temporal and causal relationship. Since cardiovascular pathology is the leading cause of mortality worldwide, the impact of quantifying these misfolded proteins in a preclinical phase could offer its greatest utility in primary prevention, with timely interventions that allow reducing the burden of these diseases in high-risk patients, as well as the associated complications: at the individual level, with improvement in quality of life, reduction in hospitalization time and mortality; and at the public level, reduction in the economic and social burden of these diseases. The clinical translation of these findings is compelling because it allows us to observe how the phenomena described in experimental models are reproduced with precision in human tissue, confirming their biological relevance and clinical applicability (Figure 8).

### 2.6. The Role of Protein Amyloid in Acute Coronary Syndrome

In the cardiovascular setting, SAA and hIAPP have emerged as biomolecules of considerable interest due to their strong and consistent association with the early onset of ACS as an acute-phase protein. SAA is rapidly upregulated in response to systemic and local inflammation, reflecting underlying inflammatory activation. However, accumulating clinical and translational evidence indicates that its significance extends well beyond that of a nonspecific marker of inflammation, positioning it as a molecule with direct implications for both diagnostic and prognostic applications. Elevated SAA levels have been repeatedly linked to the initial manifestation of ACS and to a greater probability of recurrent ischemic events, underscoring its role as a dynamic indicator of disease activity. Importantly, SAA serves not only as a diagnostic adjunct during the acute phase of ACS but also as a prognostic biomarker, capable of identifying patients at heightened risk for subsequent cardiovascular complications, including reinfarction, heart failure, and cardiovascular death [9,16]. Evidence from clinical cohorts provides robust support for the prognostic relevance of SAA. The TIMI 11A sub-study by Morrow et al. reported that among 435 patients with unstable angina and myocardial infarction, those within the highest quintile of SAA exhibited a substantially higher probability of 14-day mortality compared with patients in lower quintiles. This finding highlights the ability of SAA to function as a short-term prognostic marker, capturing patients at immediate risk of adverse outcomes [29]. Extending these observations, in a prospective cohort of 280 reperfused AMI patients, SAA concentrations in the upper quintile at 24 h (≥325 μg/dL) were significantly associated with a higher incidence of major adverse cardiovascular events (MACEs), including cardiac rupture, cardiogenic shock, subacute thrombosis, ventricular fibrillation, and pulmonary edema. These patients also exhibited reduced chronic left ventricular ejection fraction (52 ± 14% vs. 57 ± 13%; *p* = 0.03) and an increased rate of 6-month cardiovascular mortality (16% vs. 3%; *p* = 0.0003). Multivariate analysis confirmed that SAA was an independent predictor of mortality (RR 5.8; 95% CI: 1.3–27.7; *p* = 0.03), emphasizing its potential utility for early risk stratification in ACS [30].

The post-myocardial infarction period represents a critical temporal window during which the interplay between myocardial healing and adverse remodeling largely determines long-term outcomes. Persistent systemic and local inflammation during this phase plays a pivotal role in ventricular recovery, modulating processes such as scar formation, myocardial fibrosis, and the likelihood of recurrent ischemic events. Supporting this, Cederström et al. reported that three months after a first myocardial infarction in patients younger than 60 years, SAA levels remained significantly elevated relative to healthy controls (median 2.63 vs. 2.02 mg/L; *p* < 0.001). This sustained elevation reflects ongoing inflammatory activity and highlights the importance of monitoring inflammatory biomarkers during the post-infarction period [31]. In patients undergoing reperfusion therapy after acute myocardial infarction, elevated SAA levels remain a robust marker of ongoing inflammatory activity; in the study of Katayama et. al. [32], in a cohort of 433 AMI patients undergoing percutaneous coronary intervention (PCI), SAA measured at 24 h was significantly higher in those who later developed cardiac rupture (715 ± 502 vs. 206 ± 294 μg/dL; *p* < 0.0001), and using a cut-off of ≥290 μg/dL, SAA predicted rupture with an odds ratio of 8.8 (95% CI: 1.7–25.6; *p* = 0.01), demonstrating its potential as an early prognostic marker for severe post-infarction complications. Despite the general consensus on the prognostic value of SAA, some studies have reported less conclusive results [32]. While the majority of evidence supports its use as a biomarker of acute myocardial injury, contradictory to the favorable findings previously discussed, other authors have published unfavorable results that challenge the hypothesis supporting these oligomeric biomarkers. For instance, Harb et al., in a multicenter study [33] involving 1045 adults post-MI, observed that at 2–3 months follow-up, SAA did not significantly predict recurrent coronary events (HR 1.19–1.68; 95% CI not specified). Furthermore, earlier data from the large-scale European Concerted Action on Thrombosis and Disabilities (ECAT) Angina Pectoris Study by Haverkate et al. [34], which included 2121 outpatients, provided negative findings regarding SAA. The study demonstrated a global geometric mean SAA of 3.67 mg/L with no significant difference across unstable (3.72 mg/L), stable (3.74 mg/L), or atypical angina (3.42 mg/L) (*p* = 0.80). Over a 2-year follow-up recording 75 coronary events, defined as non-fatal acute myocardial infarction and sudden death, SAA failed to establish an independent association (RR 1.14 per 1 standard deviation increase; 95% CI: 0.90–1.44; *p* = 0.27) [33,34].

While these data introduce some caution regarding the universality of SAA’s predictive power, the majority of evidence supports its use as a biomarker of both acute and chronic myocardial injury [33].

### 2.7. Oligomers, Inflammation and Long-Term Cardiovascular Disease

Inflammation has emerged as a central factor in the pathogenesis of coronary artery disease, with multiple studies demonstrating that inflammatory processes play an active role in the initiation, progression, and complication of coronary atherosclerosis, contributing to plaque formation, growth, and instability, thereby highlighting the importance of inflammation and its association with oligomers. Johnson, B. et al. [35] conducted a study in 224 women referred for suspected coronary artery disease (CAD); in this analysis, SAA levels ≥10.8 mg/L were identified as an independent predictor of MACE with a 3.1-fold higher risk (HR 3.1; *p* = 0.007); furthermore, elevated SAA was significantly associated with the presence of obstructive CAD [35]. To further support the link between inflammation, SAA, and adverse cardiovascular outcomes, Ridker et al. [16] reported similar findings in a cohort of postmenopausal women where elevated SAA levels were significantly associated with major cardiovascular events in the setting of chronic inflammation; women who experienced events showed higher SAA concentrations compared with those without events (0.63 mg/dL vs. 0.52 mg/dL, *p* = 0.003) [16]. In addition to these female cohorts, novel cardiovascular risk factors and cardiac event predictors have been evaluated in other specific populations, such as female inactive systemic lupus erythematosus patients [36].

Beyond their prognostic significance, SAA, hIAPP, Aβ1-40 and Aβ1-42 oligomers have been explored as biomarkers of myocardial injury, where these molecules reflect underlying inflammatory and cardiomyocyte stress processes, offering potential for early detection of cardiac damage and monitoring disease progression. Zairis et al. [37] analyzed 519 non–ST elevation myocardial infarction (NSTEMI) patients undergoing coronary angiography and found that quantitative elevations of SAA correlated with the extent of epicardial coronary lesions; higher SAA levels were associated with an increased number of lesions (0.4 mg for 1 lesion, 2.3 mg for 2 lesions, and 4.3 mg for ≥3 lesions; *p* < 0.001) and independently predicted the presence of ≥2 complex lesions (RR 1.2 per unit; 95% CI: 1.1–1.3) [37]. SAA has also been associated with endothelial dysfunction, a key mechanism underlying systemic atherosclerosis. Narins et al. [38], in a cohort of 1045 AMI survivors, demonstrated that SAA levels were significantly elevated in patients with peripheral artery disease and clinical claudication compared to those without symptoms (0.60 mg/dL vs. 0.36 mg/dL; *p* < 0.001); this association underscores the capacity of SAA to reflect widespread endothelial impairment, which manifests not only in coronary arteries but also in peripheral vascular territories. These findings suggest that SAA may not only reflect acute myocardial injury, but also predict the burden of chronic epicardial coronary disease, and be an integrative biomarker, capturing both systemic inflammation and vascular dysfunction across multiple arterial beds [38].

### 2.8. Molecular Signatures of Inflammation in Cardiovascular Disease: The Emerging Role of SAA and Amyloid Aggregate

Inflammation is a key contributor to the pathophysiology of atherosclerosis and the development of MACE, as it promotes plaque formation, destabilization, rupture, platelet aggregation, and thrombosis [9,16]. In a prospective cohort of 40 AMI patients, Casl et al. [39] demonstrated that in patients with AMI at admission, SAA levels were substantially higher in those who subsequently developed complications, with mean values of 379 mg/L compared to 45 mg/L in patients without complications. Three days after admission, SAA concentrations exceeding 1000 mg/L were associated with a 64% incidence of complications and 36% mortality, reflecting the strong association between elevated SAA and adverse outcomes. Furthermore, an admission threshold of 100 mg/L doubled the risk of complications (sensitivity and positive predictive value 73%) and quadrupled the risk of death (sensitivity 80%), emphasizing the prognostic value of early SAA measurement in stratifying high-risk patients [39]. Ogasawara et al. [47] identified the SAA/LDL complex as an independent predictor of adverse cardiovascular events, including cardiac death, myocardial infarction, stroke, and revascularization. Specifically, each 10 μg/mL increase in the SAA/LDL complex was associated with a more than twofold higher risk of experiencing such outcomes (OR 2.32; 95% CI: 1.05–4.70). Moreover, baseline concentrations of the complex were significantly higher in patients who developed events compared to those who did not (30.60 ± 12.67 vs. 21.77 ± 7.40 μg/mL), underscoring its potential role as a predictive biomarker in cardiovascular disease [47]. Taken together, these data highlight SAA, hIAPP, Aβ1-40 and Aβ1-42 oligomers as dynamic and multifaceted biomarkers, reflecting both acute myocardial injury and ongoing post-infarction inflammation. Their measurement allows clinicians to quantify risk, interpret the magnitude of inflammatory response, and stratify patients according to their probability of developing recurrent ischemic events, heart failure, or cardiovascular death.

### 2.9. Amyloid Aβ Meets Clinical Prognosis of Adverse Cardiovascular Event

Amyolid beta is a short peptide generated by proteolytic processing of the transmembrane amyloid precursor protein (APP), which itself contributes to cell adhesion and has been implicated in hemostasis. Serum levels have been observed to increase with age and during inflammatory states, and emerging evidence suggests a potential association with ACS [46,48]. Notably, Aβ1-40 exhibits a vascular tropism, supporting the hypothesis that it may exert proinflammatory effects not only in the central nervous system but also within the walls of the carotid and coronary arteries, the aorta, and the heart itself. Regarding Aβ and AMI phenotypes, studies remain limited. Among the most relevant, Aleksova et al. [46] investigated 1119 AMI patients, comprising 68% with ST elevation myocardial infarction (STEMI) and 32% with NSTEMI, finding that a 10 pg/mL increase in Aβ1-40 was associated with a 3% higher risk of mortality (HR 1.03; 95% CI: 1.01–1.05), with a persistent signal in STEMI (HR 1.03; 95% CI: 1.01–1.05) but a non-significant signal in NSTEMI. Higher Aβ1-40 levels were also linked to older age (OR 1.13 per 5 years), chronic kidney disease (OR 4.37), HbA1c ≥39 mmol/mol (OR 1.75), and lower left ventricular ejection fraction (LVEF) (OR 1.14 per 5-point decrease). These results underscore the robust association between this biomarker and clinical outcomes, particularly early and long-term mortality.. While Aβ1-40 has garnered significant attention, other isoforms have also been evaluated. In a study by Del Moral [48], Aβ1-42 was higher in NSTEMI than STEMI patients (41.76 vs. 35.96 pg/mL; *p* = 0.001), and a cut-off of ≥38 pg/mL effectively discriminated NSTEMI cases (sensitivity 77%, specificity 74%; OR 13.184; 95% CI: 2.943–59.053) [48].

The relationship between inflammatory markers and cardiac function in acute coronary syndrome shows differences in ST-segment elevation myocardial infarction (STEMI) and in non-STEMI models [40]. Additionally, in patients with acute coronary syndromes, there is a correlation between ST-T-segment changes and markers of hemostasis [49].

### 2.10. Amyloid Oligomers: Biomarkers for Heart Failure and Metabolic Syndrome-Risk, Recurrence, and Mortality

Heart failure represents a common final stage of cardiometabolic disorders and consistently activates inflammatory pathways that overlap with metabolic syndrome. In the general population of the Rotterdam Study (n = 4156; 10.2-year follow-up), a 1 standard deviation increase in Aβ40 was associated with a 32% higher risk of incident HF (HR 1.32; 95% CI: 1.15–1.51), with a signal in men (HR 1.31; 95% CI: 1.14–1.54) but not in women (HR 1.06; 95% CI: 0.93–1.22) [50]. Translational evidence demonstrates that amylin oligomers accumulate within cardiomyocytes and are associated with both systolic and diastolic dysfunction; specifically, systolic impairment is reflected by a reduced LVEF (52.1% ± 4.8 vs. 64.3% ± 3.9; *p* < 0.05), while diastolic dysfunction is indicated by a significant decrease in early diastolic tissue velocity (E’ ~35%), suggesting impaired ventricular relaxation and increased myocardial stiffness. In addition, these structural and functional alterations are accompanied by enhanced oxidative stress and mitochondrial dysfunction (ATP decreased by ~35%; ROS increased ×3.2), which likely exacerbate myocardial contractile deficits. Collectively, these findings highlight the multifaceted impact of amylin aggregation on cardiac performance, linking metabolic dysregulation with mechanical and cellular derangements in the heart [26]. Consistent with the evidence demonstrated in humans, similar findings have also been observed in preclinical models, particularly in studies involving rats. Hall, L. et. al. [18], in obese mice, found that Aβ42 induced marked diastolic dysfunction, demonstrated by a 35% increase in E/e’, indicating impaired ventricular relaxation and elevated filling pressures. Systolic performance was also compromised, as shown by a 12% reduction in fractional shortening, reflecting diminished myocardial contractility. Importantly, these functional impairments were completely reversed following Aβ42 neutralization, highlighting the causal role of Aβ42 in cardiac dysfunction. These findings emphasize the critical influence of Aβ42 accumulation on myocardial mechanics, linking metabolic and amyloid dysregulation to functional cardiac deficits [18].

Recent research highlights hIAPP oligomers as central toxic species that contribute to pancreatic β-cell injury and extend their detrimental effects to other tissues, particularly under conditions of metabolic stress such as glucotoxicity and lipotoxicity [11,51]. In support of this, Wu et al. [19] reported elevated SAA concentrations (57.6 µg/mL by ELISA), which showed a linear correlation with age and were significantly increased in individuals with T2D (16%) and AMI (84%) [19]. Altamirano-Bustamante et al. [17] conducted observational study in pediatric and adolescent populations where circulating hIAPP oligomer levels were significantly higher in individuals with obesity and diabetes compared to healthy controls (*p* < 0.001); a diagnostic threshold of ≥3.35 μg/mL was identified (AUC 0.83), providing robust discriminative capacity with 79% sensitivity and 82% specificity. Moreover elevated hIAPP levels showed significant associations with cardiometabolic markers, suggesting that the presence of these oligomers may serve as an early indicator of cardiometabolic dysfunction risk in young individuals. In this context, SAA and hIAPP emerge as complementary molecular mediators, linking systemic inflammation and metabolic stress to vascular and myocardial pathology. The evidence suggests these mediators are not only biomarkers of early metabolic and cardiovascular perturbations, but also active participants in the pathophysiological processes bridging metabolic dysregulation and cardiovascular disease [17,25].

## 3. Discussion

Misfolded proteins can aggregate both intracellularly and extracellularly, forming toxic oligomers and amyloid fibrils that contribute to a wide spectrum of diseases, including cancer, neurodegenerative disorders such as Parkinson’s disease and Alzheimer’s disease, and chronic conditions such as obesity, T2D, metabolic syndrome, and cardiovascular disease. These conditions, classified as protein conformational diseases (PCDs) by Carrell [52], are influenced by the structural polymorphism of amyloid proteins, which may determine their degree of cellular toxicity [41].

Rather than treating heterogeneity as a methodological limitation, this review conceptualizes it as an epistemological characteristic of an emerging scientific field. The diversity of PCD, clinical endpoints, validation strategies, and implementation contexts reflects the ongoing construction of knowledge surrounding cardiovascular diseases. Consequently, an epistemic meta-analysis offers a more appropriate analytical lens than conventional statistical aggregation, enabling the examination of how evidence is produced, legitimized, and translated into clinical practice. This approach shifts the focus from the mere quantification of effects to the critical evaluation of the knowledge architectures that underpin scientific advancement in PCD.

Atherosclerotic plaques provide a clear example of how molecular misfolding contributes to pathology; plaques originate in the intima as LDL particles accumulate, undergo oxidative modifications, and acquire proinflammatory and immunogenic properties [9,51]. Progression involves extracellular matrix synthesis by smooth muscle cells to thicken the intima, while IFN-γ suppresses collagen production and macrophage-derived matrix metalloproteinases (MMPs) degrade existing collagen, weakening the fibrous cap; apoptosis of vascular cells with defective efferocytosis leads to the formation of a necrotic core, increasing the risk of plaque rupture [7,9]. Metabolic or infectious stress similarly triggers the misfolding of IAPP, SAA, amylin, and related oligomers, which provoke a maladaptive inflammatory–thrombotic response [11].

In pancreatic β cells, obesity, hyperglycemia, and inflammatory signals induce the formation of soluble oligomers (trimers to dodecamers) that disseminate systemically and interact with cellular membranes. The evidence identified in our review, indicating that the presence of IAPP is greater in patients with STEMI, and that this is associated with increased long-term mortality, suggests that these oligomers may contribute to cellular damage through multiple mechanisms. Specifically, they may disrupt calcium homeostasis, induce apoptosis, and trigger mitochondrial stress, ultimately compromising cellular integrity. In addition, these oligomers induce robust activation of innate immunity and promote the release of proinflammatory cytokines such as TNF-α, IL-1, and IL-6, thereby establishing a systemic inflammatory response (SIRS) [39,46]. Clinically, this cascade manifests as endothelial damage, increased vascular permeability, vasodilation, and hypotension, impairing tissue perfusion and facilitating progression toward organ dysfunction that may involve the pancreas, brain, or heart [47,48]. Concomitantly, activation of the coagulation pathway leads to microthrombus formation and, in severe cases, disseminated intravascular coagulation (DIC) and multiorgan damage [39,47]. During the late phase of oligomer accumulation, the coupling between inflammation and thrombosis—termed immunothrombosis—perpetuates a vicious cycle of hypoperfusion, ischemia, and tissue damage, thereby increasing the risk of adverse outcomes [9,16].

The direct deposition of hIAPP, SAA, Aβ1-40, Aβ1-42, and other misfolded oligomers may also disrupt membrane integrity. Although the toxic oligomer hypothesis remains the predominant model for amyloid-mediated toxicity, alternative mechanisms have also been proposed [42]. Small soluble oligomers are generally regarded as the most cytotoxic amyloid species, with toxicity progressively decreasing as aggregates grow into larger assemblies and mature fibrils [17,41]. However, amyloid fibrils are not completely inert and may contribute to cellular damage through mechanisms distinct from those of oligomers. In particular, fibrillar deposits can sequester surrounding cells and disrupt tissue architecture, promoting apoptotic pathways and cellular dysfunction. Thus, amyloid-associated toxicity is likely a multifactorial process in which different aggregate species exert distinct biological effects depending on their structural state and tissue context [17,43].

In the myocardium, infiltration may result in subclinical damage that, even in the absence of overt symptoms, predisposes to progressive functional deterioration [11,51]. The inflammation known to be perpetuated or promoted by IAPP, together with its increased presence in patients with vascular events, may represent a fundamental component of the clinical findings observed in these patients, which may range from heart failure with preserved ejection fraction (HFpEF) or reduced ejection fraction (HFrEF), to ischemic heart disease, arrhythmias, and cardiovascular death [38,50].

We demonstrated that given the inflammatory nature of atherosclerosis and the findings of misfolded proteins in scenarios such as myocardial infarction, misfolded proteins may intensify local inflammation and directly damage cardiomyocytes, including the promotion of apoptosis in ischemic cells. In relation to this, some studies show that elevated levels of serum amyloid markers such as SAA correlate with adverse outcomes, including myocardial infarction, cardiac rupture, and peripheral arterial disease [31,33].

Amplified inflammation and endothelial damage destabilize atherosclerotic plaques and impair microvascular function, favoring acute coronary syndromes, sustained myocardial injury, and reinfarction [9,16]. Importantly, these pathogenic processes extend beyond the heart; through mechanisms of inflammation, endothelial damage, and immunothrombosis, the accumulation of misfolded proteins generates a systemic phenotype encompassing diabetes mellitus, neuropsychiatric disorders, cancer, retinopathy, micro- and macroangiopathy, neuronal dysfunction, and cognitive impairment. In advanced stages of oligomer accumulation, endothelial damage and multiorgan injury predominate, accompanied by immunoparalysis characterized by lymphocyte apoptosis, reduced expression of HLA and IL-12, and increased IL-10, consistent with a state of immunosuppression [29,30].

Given the inflammatory nature of atherosclerosis, these misfolded proteins may intensify local inflammation and directly damage cardiomyocytes, including the promotion of apoptosis in ischemic cells. Clinical studies support this, showing that elevated levels of serum amyloid markers such as SAA correlate with adverse outcomes, including myocardial infarction, cardiac rupture, and peripheral arterial disease [26,27]. The interaction between systemic metabolic disorders—particularly T2D and obesity—and cardiac amyloid deposition highlights hIAPP as a mechanistic driver and a potential biomarker of cardiovascular risk. Far from constituting a nonspecific inflammatory biomarker, SAA emerges as an active mediator capable of modulating multiple pathogenic axes relevant to the progression of atherosclerotic disease and the clinical evolution of acute coronary syndromes. From this pathophysiological perspective, the association between SAA levels and cardiovascular outcomes should be interpreted within an integrative model that links systemic inflammation, endothelial dysfunction, immunothrombosis, and protein misfolding [35]. In this sense, it has been proposed that its expression not only reflects the intensity of the acute-phase response, but also the persistence of a maladaptive inflammatory phenotype that confers vascular vulnerability and ongoing myocardial damage [38]. Therefore, SAA could be consolidated as a key tool in the longitudinal monitoring of the cardiovascular patient; studies in which serial measurements have been made would allow evaluation of the response to therapeutic interventions, identification of persistent residual inflammation, and early detection of unfavorable clinical trajectories.

In the clinical context, particularly in acute coronary syndrome, the incorporation of SAA could redefine risk stratification by overcoming the limitations of traditional biomarkers, since while troponins capture myocardial necrosis and C-reactive protein provides a relatively static estimate of inflammation, SAA emerges as a biomarker with more sensitive and dynamic kinetics, closely related to activation of the innate immune system and instability of the atherosclerotic plaque [16,35]. Accordingly, the behavior of this biomarker allows the identification of a subgroup of patients with residual inflammatory risk characterized by a greater propensity for adverse events even in apparently stable phases or in the presence of non-significantly elevated conventional biomarkers. The identification of this high-risk inflammatory phenotype opens the door to individualized therapeutic intensification strategies; in this scenario, elevated SAA levels could justify the adoption of more aggressive and earlier interventions in this pathology with a high global burden of morbidity and mortality, enabling the implementation of processes that include optimization of medical therapy, primary prevention, and consideration of therapies targeting inflammation. This approach represents a relevant conceptual shift: from treatment focused exclusively on reperfusion to primary prevention, with profound clinical implications, as it suggests that a relevant proportion of cardiovascular risk is not being captured by current algorithms.

SAA, IAPP, and amyloid oligomers acquire an even more relevant dimension when analyzed together as emerging biomarkers. A hypothesis can be proposed in which protein misfolding acts as a central mechanism that connects metabolism, inflammation, and immunity, given that these misfolded proteins not only accumulate, but also interact with cellular membranes, alter calcium homeostasis, induce mitochondrial stress, and promote apoptosis, amplifying tissue damage, favoring inflammasome activation, and perpetuating a state of chronic low-grade inflammation with episodes of acute decompensation [17,25]. This mechanistic understanding could have direct implications for the identification of new therapeutic targets, in contrast to traditional approaches focused on classical risk factors, allowing intervention at earlier and more fundamental stages of disease. Among the potential strategies are the inhibition of oligomer formation and aggregation, the blockade of their interaction with cellular structures, and the modulation of specific inflammatory pathways as the central part [39,47]; these approaches not only aim to mitigate damage, but to modify the natural course of the disease. The accumulated experience in diseases such as Alzheimer’s disease has demonstrated the feasibility of strategies targeting misfolded proteins, such as monoclonal antibodies and conformational stabilizers. Extrapolating oligomer-based strategies and hypotheses as biomarkers to the cardiovascular field, these could represent an innovative frontier with the potential to impact the progression of atherosclerosis, plaque stability, and myocardial remodeling [41,52]. In this context, the development of targeted anti-inflammatory therapies becomes particularly relevant, as the possibility of selecting patients based on their biomolecular profile—for example, elevated SAA levels—would allow optimization of primary prevention therapy, ranging from lifestyle modification to agents such as IL-1 or IL-6 inhibitors, maximizing clinical benefit and minimizing risks [31,48].

With the above findings, it is proposed that understanding the pathophysiology of these biomarkers, encompassing protein misfolding to cellular damage and the clinical expression of disease, aligns with the principles of personalized medicine, where biomarker-based stratification allows therapy to be tailored to the individual characteristics of the patient. Ultimately, this translates from the molecular perspective to clinical representation with a significant reduction in major cardiovascular events, contributing to the prevention of complications and disease progression. This is particularly relevant in subclinical phases or in patients with stable disease, where early intervention could modify the natural trajectory toward major clinical events, directly impacting the reduction in the global burden of morbidity and mortality and healthcare system costs. Taken together, the available evidence supports the notion and hypothesis that SAA and associated biomarkers should not be considered solely diagnostic or prognostic tools, but active components of a complex pathophysiological network integrating inflammation, metabolism, and thrombosis. Their incorporation into clinical practice would not only improve risk stratification and therapeutic decision-making, but also drive the development of more precise targeted strategies, marking a transition toward a truly mechanistic and personalized model of cardiology.

The relationship between metabolic disorders and cardiovascular disease is underscored by the systemic impact of inflammatory and misfolded biomarkers. The concept of metabolic syndrome provides a framework for understanding these overlapping risks in older adult populations [20]. In conditions such as obesity and diabetes, elevated circulating amyloid concentrations have been shown to promote vascular dysfunction [44]. This aligns with findings demonstrating that inflammatory markers, including serum amyloid A, C-reactive protein, and fibrinogen, are associated with prevalent coronary heart disease [45]. Following an acute coronary syndrome, systemic inflammation continues to drive adverse outcomes, with inflammatory biomarkers serving as indicators for mortality and recurrent nonfatal events [53]. Furthermore, other inflammatory proteins like plasma pentraxin 3, while not always predicting future coronary events, reflect the underlying metabolic disorders in patients with coronary artery disease [21].

### The Heart as a Primary Target Organ in Protein Conformational Diseases

**Figure 9:**
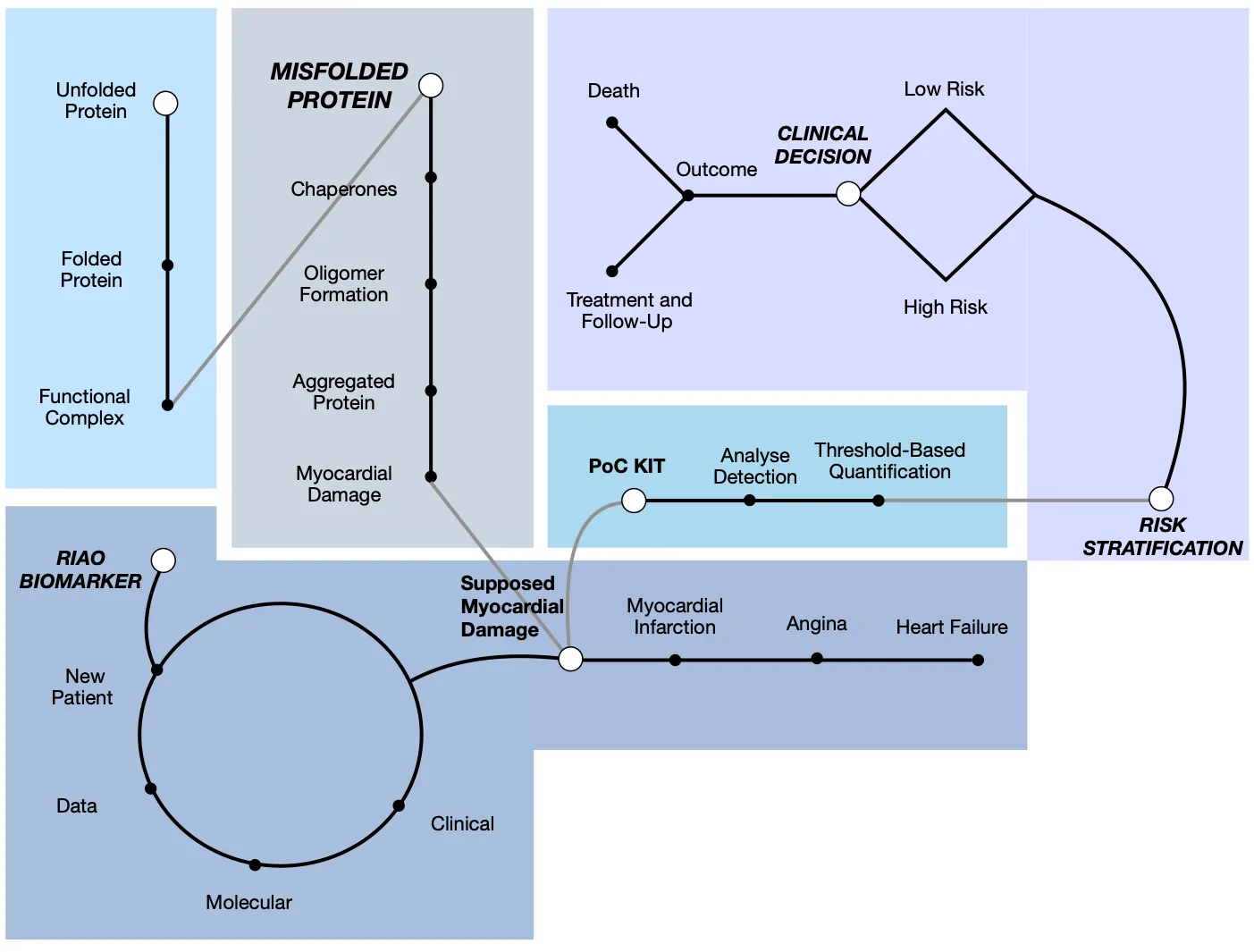
Roadmap for integrating misfolded protein biomarkers into clinical decision-making. Conceptual diagram illustrating how emerging evidence on misfolded proteins, including IAPP, SAA, and Aβ, can be translated into risk stratification, diagnostic algorithms, and individualized management strategies for cardiovascular [46]. The figure illustrates a conceptual model of a paradigm shift in cardiovascular disease centered on the role of amyloid oligomers as early biomarkers of myocardial damage. It organizes the process into a logical sequence that includes problem identification, strategy design, application of a new diagnostic approach, and results analysis. This strategy enables progression toward concrete clinical applications, such as early detection, risk stratification, and therapeutic decision-making. The diagram is divided into five color-coded sections representing distinct phases of the translational workflow: light blue indicates normal protein folding and physiological function; grey represents the pathological protein misfolding and aggregation pathway; dark blue highlights the biomarker identification process; cyan denotes the diagnostic phase using Point-of-Care (PoC) testing; and violet outlines the clinical outcomes, decision-making, and risk stratification.

Traditionally, PCDs—a foundational concept established by Carrell (2005) [52]—have been approached in a compartmentalized manner, largely restricted to neurodegenerative disorders (such as Alzheimer’s disease) or localized metabolic pathologies, such as pancreatic islet amyloidosis mediated by hIAPP in T2D. However, the integrative translational evidence compiled in our epistemic analysis demands a fundamental paradigm shift: conceiving the cardiovascular system—and the myocardium in particular—as a primary systemic target organ for the deposition and action of circulating proteotoxic species [15,26] (Figure 9).

Because the myocardium operates under continuous mechanical stress and possesses exceptionally high bioenergetic demands, it is uniquely vulnerable to the circulating load of soluble, non-native oligomers. Beyond viewing cardiac involvement as mere collateral damage secondary to local ischemia and chronic low-grade inflammation of atherosclerosis [9], the synthesized literature supports the concept that the systemic dissemination of circulating amyloid oligomers generates a multi-hit molecular microenvironment.

From a mechanistic perspective, these circulating aggregates—originating from hypermetabolic or inflamed organs such as the pancreas, adipose tissue, or the liver—manage to cross the coronary endothelium and bind directly to the sarcolemma of cardiomyocytes [31]. As demonstrated by the experimental models of Despa et al. [26,28] and Hall et al. [18], the interaction of hIAPP and Aβ oligomers with the cell membrane triggers a pathological cascade characterized by:

1. Calcium homeostasis alteration and contractile dysfunction: A sarcolemmal Ca 2+ leak and a decreased capacity of the sarcoplasmic reticulum occur, translating clinically into reduced LVEF and impaired diastolic relaxation (E/e ‘), leading to HFpEF or HFrEF [26,50].
2. Oxidative stress and mitochondrial collapse: Oligomer accumulation dramatically increases reactive oxygen species (ROS) production (up to 3.2-fold) and decreases adenosine triphosphate (ATP) levels (by approximately 35%), disorganizing mitochondrial architecture and promoting cardiomyocyte apoptosis [18].
3. A gradient of structural complexity and clinical progression: As Despa et al. [26,28] observed in human hearts, small soluble oligomers (trimers and tetramers at 12–16 kDa) are identified early in prediabetic states or non-failing hearts of overweight individuals, whereas higher-molecular-weight aggregates (32–64 kDa and greater than 150 kDa) are detected exclusively in hearts with end-stage diabetic heart failure. This outlines a pathological gradient accompanying the clinical course of myocardial dysfunction.

Finally, compounding this network is the capacity for co-aggregation or amyloid cross-seeding, where distinct misfolded proteins interact synergistically to amplify cytotoxicity. This entire process demonstrates that cardiac impairment in conformational diseases is not an isolated event, but the result of a complex systemic network wherein the myocardium acts as a susceptible target for cumulative proteotoxic damage long before overt clinical decompensation manifests [13].

Several methodological limitations of this review should be acknowledged. The term RIAO specifically refers to hIAPP oligomers, while other evaluated assemblies—such as SAA aggregates and amyloid-beta (Aβ1-40 and Aβ1-42) oligomers—comprise distinct, separate categories of amyloid proteins. Furthermore, the specific nomenclature of RIAO and several foundational studies on these circulating oligomers originate primarily from our research group. While these investigations provide critical quantitative insights into conformational proteopathies and metabolic-cardiovascular links, readers should consider this as a potential source of institutional citation concentration and self-selection bias.

## 4. Materials and Methods

Following the PRISMA guidelines, the present review systematically identified, screened, and evaluated the available evidence relevant to the research question. This systematic review (SR) was developed using a modified PIO framework (Population/Intervention/Outcome) and adhered to the PRISMA methodology [16]. The search was conducted across three electronic databases: PubMed, BIREME, and Web of Science. These three databases were selected for their strong coverage of biomedical and Latin American literature; however, this choice may have limited the retrieval of studies indexed exclusively in other databases.

### 4.1. Search Strategy

This systematic review, conducted from June 2025 to October 2025, was carried out using the following search strategy: ((“Heart attack”) OR (“Myocardial infarction”) OR (“Cardiac dysfunction”) OR (“Endothelial dysfunction”)) AND ((“Amylin”) OR (“RIAO” ) OR (“Oligomers”) OR (“Islet amyloid polypeptide”) OR (“Amyloid”) OR (“Protein misfolding”) OR (“Protein aggregation”) OR (“Protein conformational diseases”) OR (“Conformational diseases”)) AND ((“Inflammation”) OR (“Heart failure”) OR (“Mortality”)).

This strategy was applied to the title, abstract, and keywords (or equivalent fields in each database) to ensure comprehensive retrieval.

Eligibility Criteria

To ensure clarity and minimize bias, study selection followed the PRISMA guidelines and consisted of four phases:

• Phase 1: All search results were compiled, noting the number of articles retrieved from each database and the total combined.
• Phase 2: A first screening was conducted using the Mendeley web tool to remove duplicate studies.
• Phase 3: Titles and abstracts were reviewed to exclude studies irrelevant to the research question. Articles were included if they were in English or Spanish and had a title, abstract, or keywords relevant to the topic. Studies in other languages, as well as book chapters, theses, and conference abstracts, were excluded. Selected articles were compiled in a preliminary table.
• Phase 4: A final screening assessed the methodological quality of each full article using five criteria:
  (a) Clearly defined research objectives;
  (b) Alignment between objectives and research question;
  (c) Appropriate methodology;
  (d) Clear definitions of key terms;
  (e) Results consistent with objectives.

Three independent reviewers (FACG, DRZ, MMAB) analyzed the titles and abstracts of all retrieved records to identify studies that met the predefined eligibility criteria for subsequent full-text evaluation. A third assessment stage was then applied to the selected articles to determine their suitability based on methodological quality, evaluated using the five previously described items. Studies achieving a methodological quality score greater than 80% were considered adequate and therefore fulfilled the eligibility criteria for inclusion in this review. For the systematic review process, relevant information was extracted from each article using the PIO framework, focusing on the research question to ensure consistent synthesis across studies.

### 4.2. Methodological Quality and Risk Bias

Methodological quality and risk of bias were assessed using the Newcastle–Ottawa Scale adapted for cross-sectional association studies (NOS-xs), the Newcastle–Ottawa Scale (NOS) for cohort and case–control studies, and SYRCLE’s Risk of Bias tool for animal studies.

### 4.3. Mind Maps Approach and Organization

We followed a mind map approach similar to that of Chilaca and et al. [23], organizing the information into two complementary components: what was reported and how it was conducted. The first component summarizes the main findings of each study, including the research objective, disease context, analytical outcomes, and key results. The second component captures the methodological aspects, such as sample source and characteristics, experimental techniques, data acquisition and processing procedures, analytical approaches, and other information supporting the reported findings. This structure facilitates both the interpretation of the results and the comparison of methodological differences across studies.

### 4.4. Epistemic Meta-Analytical Framework: Exploring the Role of Amyloid Oligomer in Myocardial Damage

Given the conceptual, methodological, and clinical heterogeneity characterizing research on myocardial damage as a PCD, a conventional statistical meta-analysis was not considered appropriate. Substantial variability across studies in intervention design, outcome definitions, follow-up duration, and reporting practices limits the comparability required for quantitative pooling and effect-size estimation. To address these challenges, this review adopted an epistemic meta-analytical framework. This term and analytical approach were previously introduced by our group in Chilaca-Rosas et al. [23], who applied it to synthesize heterogeneous evidence on AI-radiomics integration for glioma progression.

Unlike traditional meta-analysis, which primarily aims to statistically aggregate homogeneous quantitative evidence, epistemic meta-analysis seeks to integrate and critically examine the knowledge structures underlying a body of research [54,55]. This approach encompasses not only empirical findings but also the theoretical assumptions, methodological choices, validation strategies, and clinical translation pathways that shape scientific evidence. Epistemic meta-analysis is particularly relevant in rapidly evolving fields where innovation frequently outpaces methodological standardization. Such diversity generates a fragmented evidence landscape in which direct statistical comparisons may obscure rather than clarify the state of knowledge [22,23,54,55].

Accordingly, the present review synthesized evidence through an integrative assessment of methodological rigor, epistemological coherence, technological approaches, and clinical applicability. Rather than focusing exclusively on pooled effect sizes, this framework evaluated the directional consistency of reported outcomes, including amyloid oligomers, outcomes, predictive performance, and decision-support effectiveness. The objective was to identify convergent patterns across studies while acknowledging the contextual factors that influence observed outcomes.

Furthermore, this epistemic perspective recognizes that knowledge production extends beyond quantitative measurements and encompasses the interpretation, contextualization, and meaning of scientific findings. By examining how evidence is generated, validated, and translated into clinical practice, this methodology provides a deeper understanding of both strengths and limitations of the current literature.

The purpose of this epistemic integration is to establish a coherent interpretative framework to understand the evolving role of myocardial damage management. Through the identification of methodological trends, recurring challenges, research gaps, and emerging areas of consensus, this analysis contributes to a more comprehensive appraisal of the field. Moreover, it supports future efforts toward harmonization of outcome measures, standardization of reporting practices, and the development of sufficiently robust evidence bases capable of supporting future quantitative meta-analyses.

For an article to be included in the epistemic meta-analysis, it was required to be an original research study published in an indexed scientific journal, available in full text in its original PDF version, and directly relevant to the research question regarding amyloid oligomers—particularly SAA and Aβ1-40—in the context of myocardial damage. Studies conducted in human populations, animal models, and in vitro settings were all considered eligible. Review articles, letters to the editor, abstracts, and viewpoints were excluded. No formal numerical scoring instrument was applied; rather, eligibility was determined through full-text evaluation against the established inclusion criteria by the research team, and all articles that fulfilled these criteria were incorporated into the analysis. The final corpus comprised 30 articles.

This study was conducted by a cross-functional team comprising medical doctors, biomedical researchers specializing in metabolic disease and cardiology, and a physical engineer with experience in data analysis, whose combined expertise allowed for a rigorous and integrative interpretation of the evidence across disciplines.

## 5. Limitations

This systematic review has several limitations that should be considered when interpreting its findings. First, the search was restricted to three databases (PubMed, BIREME, and Web of Science); databases such as Embase, Scopus, and the Cochrane Library, as well as gray literature sources, were not searched, which may have excluded additional relevant studies. Second, eligibility was limited to articles published in English or Spanish, introducing a potential language bias, as relevant evidence published in other languages may have been omitted.

## 6. Conclusions

**Figure 10:**
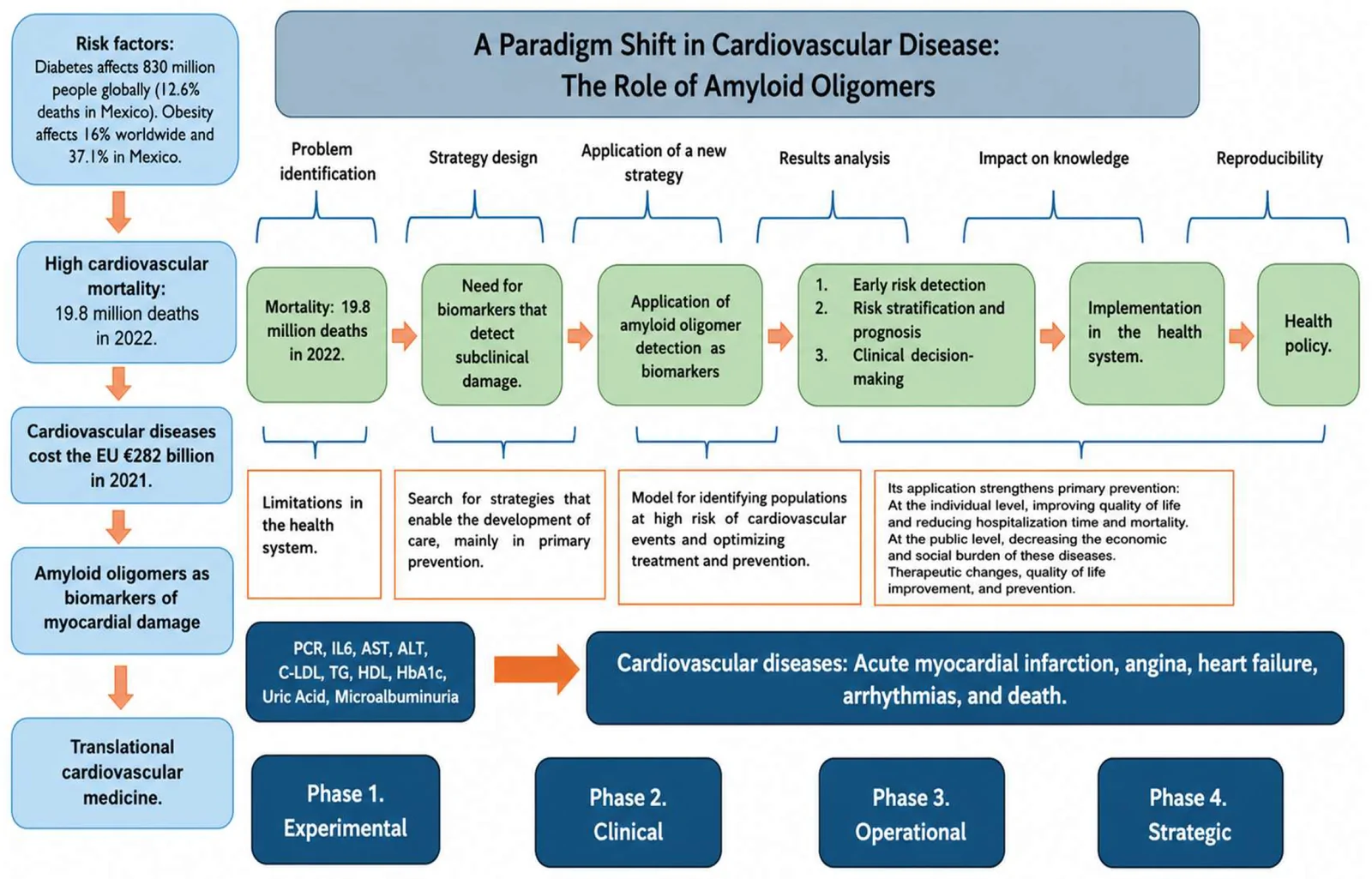
Translational model of amyloid oligomers in cardiovascular disease: from molecular mechanisms to clinical application. The figure illustrates a conceptual model of a paradigm shift in cardiovascular disease centered on the role of amyloid oligomers as early biomarkers of myocardial damage. It organizes the process into a logical sequence that includes problem identification, strategy design, application of a new diagnostic approach, and results analysis. This strategy enables progression toward concrete clinical applications, such as early detection, risk stratification, and therapeutic decision-making [36].

The central objective of this research was to determine whether the accumulation of amyloid oligomers in the cardiac cell is greater in patients with myocardial damage. Based on the presented evidence, it can be concluded that amyloid oligomers are present in greater abundance in patients with myocardial dysfunction, and that their quantity and structural complexity increase as cardiac damage progresses. This approach is relevant because throughout the reviewed evidence, key findings have emerged that support their translational potential. Among the most important findings is the possibility of early detection, since the identification of amyloid oligomers at initial stages could become a tool to recognize individuals with high cardiovascular risk even before the evident clinical manifestation. This aspect not only broadens the diagnostic window but also opens the door to closer surveillance and the application of timely preventive measures. With regard to the clinical advantage, the detection of these oligomers makes it possible to guide interventions aimed at reducing chronic inflammation, improving metabolic control, and preventing immunothrombosis; these are all mechanisms closely linked to the progression of myocardial damage and the occurrence of acute events. This ability to intervene at earlier phases turns oligomers into a biomarker with a practical value that transcends the merely experimental (Figure 10).

The impact is also reflected in the field of primary prevention, since monitoring these misfolded proteins could facilitate the implementation of strategies to avoid the occurrence of the first cardiovascular event. This has a multiplying effect on public health, since delaying or preventing the onset of cardiovascular disease significantly reduces the future burden of complications. A special mention should be made of the high-cardiometabolic-risk population, which includes patients with obesity, T2D, chronic kidney disease (CKD), previous CVD or systemic lupus erythematosus, where the determination of amyloid oligomers could serve as a risk stratification tool, allowing interventions to be personalized and the intensity of clinical follow-up to be adapted according to the degree of alteration detected. Finally, the strategic value of this line of research is expressed at both the individual and collective levels. For the patient, the early detection and management of amyloid oligomers translate into better quality of life, reduction in hospitalization time, and lower mortality. At the population level, the prevention of cardiovascular complications represents a substantial decrease in the economic and social burden associated with CVD, contributing to a more sustainable health system.

Taken together, these findings consolidate the hypothesis that amyloid oligomers are not only markers of damage, but also active and strategic mediators in the course of cardiovascular disease, capable of transforming the clinical approach toward a truly translational model, from molecular biology to clinical practice.

**Figure 11:**
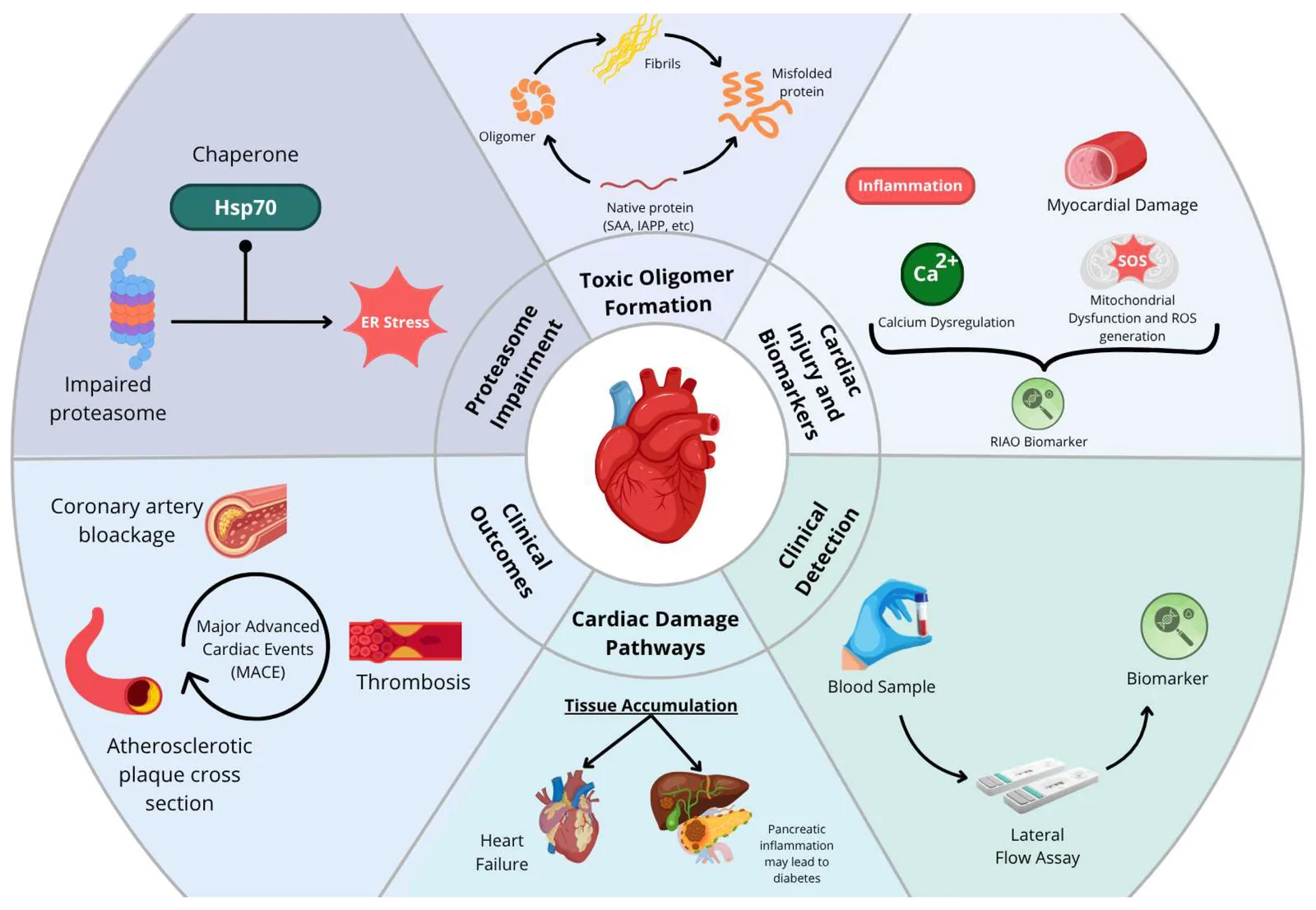
Pathophysiological cascade of protein misfolding and oligomer-induced cardiac damage. This figure illustrates how impaired proteasome function, driven by factors such as aging, inflammation, metabolic stress, and reduced proteostasis capacity, promotes protein aggregation and the misfolding of native proteins like IAPP and SAA. These misfolded proteins form toxic oligomers that circulate in the bloodstream, triggering direct tissue damage—leading to diabetes, arrhythmias, and heart failure—and amplifying inflammatory responses that destabilize plaques and cause myocardial injury. Circulating IAPP serves as an early biomarker of toxic oligomer-induced cardiac damage.

Although current evidence remains limited, further research is needed to explore the behavior of these proteins across different stages of ischemic disease and metabolic syndrome progression. Nevertheless, this systematic review demonstrates a consistent relationship between oligomers and cardiovascular pathology, supporting their role as early biomarkers that are potentially detectable before traditional markers of myocardial injury or inflammation. These insights could enable more personalized treatment strategies, refined monitoring, and optimized cardiometabolic targets, revealing novel diagnostic and therapeutic opportunities at the intersection of cardiology and metabolic medicine (Figure 11).

## Data Availability

No new primary data were created or analyzed in this study. Data sharing is not applicable to this article as all data were obtained from the previously published literature cited in the text.

## Supplementary Materials

The following supporting information can be downloaded at: https://www.mdpi.com/article/10.3390/ijms27177956/s1.

## Abbreviations

The following abbreviations are used in this manuscript:

ACS	Acute coronary syndrome
AMI	Acute myocardial infarction
CAD	Coronary artery disease
HF	Heart failure
HFpEF	Heart failure with preserved ejection fraction
HFrEF	Heart failure with reduced ejection fraction
MACE	Major adverse cardiac events
NSTEMI	Non-ST elevation myocardial infarction
STEMI	ST elevation myocardial infarction
PCI	Percutaneous coronary intervention
T2D	Type 2 diabetes mellitus
UA	Unstable angina
