# Supplementary material for: Circulating Amyloid and Misfolded Biomarkers in Early Myocardial Injury: A Systematic Review and Epistemic Meta-Analysis
Source: Int J Mol Sci. 2026 Sep 7;27(17):7956. doi: 10.3390/ijms27177956 (PMC13566443; doi:10.3390/ijms27177956)
Supplement: Supplementary file 1 [file ijms-27-07956-s001.zip › Table S1. Main characteristics of included studies. Summary of the 30 studies included in the systematic review and meta-analysis..pdf]

Table S1. Main characteristics of included studies. Summary of the 30 studies included in the systematic review and meta-analysis.

Titles and authors	Population	Intervention	Comparison	Outcome	Amiloid	Other
Alesksova, A. Italy. 2025 Amyloid β1- 40 Predicts Long- Term Mortality Rate in Patients With Acute Myocardial Infarction (MI)	The study population consisted of patients who suffered an acute MI. Number of participants: 1,119 consecutive patients. Average age: 67.4 years. Sex: 72.2% men. Diagnosis: Included both patients with ST-segment elevation myocardial infarction (STEMI), 68% of the cohort) and non-ST-segment elevation myocardial infarction (NSTEMI).	The main exposure was the plasma level of the isoform Amyloid β1-40 (Aβ1-40).<br><br>Measurement: A single measurement of Aβ1-40 was performed from a blood sample.<br>Timing: The sample was taken within the first 8 hours after hospital admission for acute myocardial infarction.	The study conducted comparisons in several ways to evaluate the prognostic value of Aβ1-40: Levels above vs. below the median: The mortality rate of patients with Aβ1-40 levels above the group median (86.9 pg/mL) was compared to those with levels below this value. Analysis as a continuous variable: In regression models, Aβ1-40 was analyzed as a continuous predictor, assessing the increase in risk per unit increase in its concentration. Deceased vs. survivors: Mean Aβ1-40 levels were compared between the group of patients who died during follow-up and those who survived.	The primary outcome measured was all-cause mortality over a long-term follow-up period.<br><br>Follow-up: The median follow-up was 57 months.<br>Events: During this period, 193 patients (17.2% of the cohort) died.	Mortality risk (Hazard Ratio - HR): In multivariable analysis, Aβ1-40 was an independent predictor of death.<br><br>In the total cohort (STEMI + NSTEMI): For every 10 pg/mL increase in Aβ1-40, the risk of death increased by 3% (HR: 1.03; 95% CI: 1.01–1.05).<br>In the STEMI subgroup: The association remained strong and significant, with an HR of 1.03 (95% CI: 1.01–1.05) per 10 pg/mL increase.<br>In the NSTEMI subgroup: No significant association was found (p = 0.17), which the authors attribute to the smaller size of this subgroup and a low event rate.<br>Study design: Cohort study. Quantification method: Aβ1-40 levels were measured using a sandwich ELISA method.	Advanced age: For every 5-year increase, the odds of having high Aβ1-40 levels increased (Odds Ratio [OR]: 1.13; 95% CI: 1.06–1.21).<br><br>Chronic Kidney Disease (CKD): Patients with an estimated glomerular filtration rate (eGFR) <60 mL/min had a much higher likelihood of elevated levels (OR: 4.37; 95% CI: 2.14–8.92).<br><br>Diabetes (Glycated Hemoglobin): An HbA1c level ≥ 39 mmol/mol also increased the likelihood (OR: 1.75; 95% CI: 1.25–2.46).<br><br>Reduced Left Ventricular Ejection Fraction (LVEF): For every 5 percentage point decrease in LVEF, the odds of having elevated Aβ1-40 increased (OR: 1.14; 95% CI: 1.06–1.24).
Casl, M. Et. Al. 1995. Croatia. Serum amyloid A protein in patients with acute myocardial infarction	Participants: The study included 40 patients diagnosed with acute myocardial infarction. Admission: All patients were admitted to the hospital between 6 and 12 hours after symptom onset. Groups: Patients were divided into two groups for analysis: Uncomplicated group: 29 patients. Complicated group: 11 patients, of whom 5 had a fatal outcome.	Exposure: The main exposure was the concentration of Serum Amyloid A protein in the blood. Measurement: SAA levels were measured daily from hospital admission and for the following nine consecutive days. The measurement was performed using a micro-ELISA assay.	The study performed key comparisons to evaluate the prognostic value of SAA: Patients with complications vs. without complications: Mean SAA levels were compared between the group that developed post-infarction complications and the group that did not. Admission cutoff: An SAA level of 100 mg/L at admission was used as a reference point to assess risk. Patients with levels above this threshold were compared to those below it. Cutoff at day 3: The rates of complications and mortality were compared among patients grouped by different SAA concentration ranges three days after the infarction (<500 mg/L, 500–1000 mg/L, and >1000 mg/L).	Main outcomes measured were post-infarction complications and mortality rate<br>Results at 3 days post-infarction:<br>Patients with SAA > 1000 mg/L had a 64% complication rate and a 36% mortality rate.	Main outcomes measured were post-infarction complications and mortality rate SAA levels at admission: Patients with complications: Mean SAA level of 379 mg/L. Patients without complications: Mean SAA level of 45 mg/L. Predictive value of SAA > 100 mg/L at admission: For Complications: Showed a sensitivity and predictive value of 73%. Patients above this threshold had twice the risk of developing complications. For Mortality: Showed a sensitivity of 80% for early prediction of a fatal outcome. Patients above this threshold had four times the risk of a fatal outcome.<br>SAA levels in patients with fatal outcome: At admission: Mean SAA level was 404 mg/L. Three days later: Mean SAA level rose to 2310 mg/L.<br>This prospective cohort study measured serum amyloid A levels using a micro-ELISA (enzyme-linked immunosorbent assay)."	Using a level of 100 mg/L on admission as a reference value gave a reasonable sensitivity and predictive value for complications and a very good sensitivity (80%) for early prediction of fatal outcome.
Cederström, S. Et al., 2024. Suceia. Inflammatory biomarkers and long-term outcome in young patients three months after a first myocardial infarction	The study population consisted of 382 patients (referred to as "cases") under the age of 60 who had experienced a first myocardial infarction. They were compared with a control group of 383 healthy individuals, matched by age and sex, from the same geographic area. The characteristics of the cases included a mean age of 52.4 years and 81.9% were male.	The intervention was the measurement of the inflammatory biomarker Serum Amyloid A levels in blood samples. The samples from patients who had experienced an MI were collected three months after the acute event.	Death, heart failure (HF) or MI hospitalization.<br>Cases (382)were patients under 60 years of age, and controls (n = 383) were age- and sex-matched individuals.	Three months after the MI, patients (cases) showed significantly higher SAA levels compared to the control group.	The median SAA level in the cases was 2.63 mg/L (interquartile range [IQR]: 1.61–4.33), while in the controls it was 2.02 mg/L (IQR: 1.30–3.00), with a p-value < 0.001.<br>Prospective cohort design with a matched control group.<br>SAA was measured using particle-enhanced immunonephelometry.	IL-6 (comparing the highest tertile vs. the lowest):<br>Composite Outcome (Death, HF, or MI): Risk was 1.91 times higher (HR 1.91, 95% CI: 1.31–2.79). All-Cause Mortality: Risk was 2.38 times higher (HR 2.38, 95% CI: 1.42–3.98).<br>Hospitalization for Heart Failure: Risk was 2.70 times higher (HR 2.70, 95% CI: 1.32–5.50).<br>IL-18 (comparing the highest tertile vs. the lowest): Hospitalization for Recurrent Myocardial Infarction: After adjusting for risk factors, the risk was 2.31 times higher (HR 2.31, 95% CI: 1.08–4.91).
Del Moral, L. Et al., 2024. México Correlation of Plasmatic Amyloid Beta Peptides (Aβ-40, Aβ-42) with Myocardial Injury and Inflammatory Biomarkers in Acute Coronary Syndrome	The study included 65 patients with acute coronary syndrome (ACS) admitted to the coronary care unit at the National Institute of Cardiology Ignacio Chávez in Mexico City. Patients were recruited within the first 12 hours after symptom onset, between February 2022 and May 2023. The population was divided into two groups: 34	The main intervention was the measurement of plasma levels of beta-amyloid peptides Aβ-40 and Aβ-42 from peripheral blood samples. These measurements were performed using high-sensitivity ELISA kits (High-Sensitivity Human Amyloid Aβ-42 and Aβ-40 ELISA Kit). The Aβ-42/40 ratio was also calculated.	The study conducted several key comparisons:<br><br>Between types of infarction: Levels of Aβ-40 and Aβ-42 were compared between the STEMI and NSTEMI patient groups.<br>Correlation with biomarkers: The correlation of amyloid peptide levels with	Aβ-42 levels in NSTEMI vs. STEMI: Patients with NSTEMI had significantly higher plasma levels of Aβ-42 compared to those with STEMI (median of 41.76 pg/mL vs. 35.96 pg/mL; p = 0.001). Aβ-40 levels in NSTEMI vs. STEMI: No statistically significant differences were found in Aβ-40 levels between the two groups (p = 0.472).	An Aβ-42 level ≥ 38 pg/mL was identified as the optimal cutoff point to distinguish patients with NSTEMI from those with STEMI, with a sensitivity of 77% and a specificity of 74%. Patients with Aβ-42 levels above this threshold had more than a 12-fold increased risk of presenting with NSTEMI (Odds Ratio = 13.184).Aβ-42 ≥ 38	Correlation with NT-proBNP: A positive and significant correlation was found between NT-proBNP levels and both peptides: Aβ-42 (Rho = 0.417, p = 0.001) and Aβ-40 (Rho = 0.374, p = 0.002).<br>Correlation with age: Both Aβ-42 and Aβ-40 showed a positive and significant correlation with patient age (p = 0.014 and p = 0.001, respectively).

	patients with ST-segment elevation myocardial infarction, with a mean age of 58 years. 31 patients with non–ST-segment elevation myocardial infarction, with a mean age of 60 years.		biomarkers of myocardial injury (high-sensitivity troponin I [hs-cTnI], NT-proBNP) and inflammation (high-sensitivity C-reactive protein [hs-CRP]) was assessed.		pg/mL OR 13.184 IC95(2.943–59.053).<br>The study was a cross-sectional, observational, and correlational single-center study. Beta-amyloid peptides Aβ-40 and Aβ-42 were measured using high-sensitivity ELISA kits (High-Sensitivity Human Amyloid Aβ-42 ELISA and High-Sensitivity Human Amyloid Aβ-40 ELISA Kit).	
Di stefano, R. Et. Al. 2009. Italy. Inflammatory markers and cardiac function in acute coronary syndrome: Difference in STEMI and in NSTEMI models	The study population consisted of 40 consecutive patients with acute coronary syndrome admitted to the University of Pisa Institute, Italy.<br>Patients were recruited within the first 12 hours after symptom onset.<br>The population was divided into two groups:<br>23 patients with STEMI, with a mean age of 61.7 years.<br>17 patients with non–ST-segment elevation myocardial infarction , with a mean age of 65.6 years.	The intervention was the measurement of Serum Amyloid A levels in the patients' blood.<br><br>Measurements were taken at three different time points:<br><br>At admission (t0).<br>At 7 days (t1).<br>At 30 days (t2).	The study conducted several key comparisons related to SAA: Between infarction types: SAA levels were compared between the STEMI and NSTEMI patient groups at the different measurement time points.<br>Correlation with cardiac function: The correlation between SAA levels at admission and cardiac function parameters measured by echocardiography, such as ejection fraction and wall motion score index (WMSI), was evaluated.<br>Temporal kinetics: The evolution of SAA levels over the 30 days was analyzed to determine whether their behavior (kinetics) differed between the STEMI and NSTEMI groups.	The main results related to SAA were:<br>Levels at admission: There was no statistically significant difference in SAA levels between the STEMI and NSTEMI groups at admission ($p = 0.061$). The median level in the STEMI group was 55.3 mg/L, while in the NSTEMI group it was 7.2 mg/L.	Regarding the inflammation kinetics, a difference was observed in the two groups only for WBC ($p < 0.05$) and SAA ($p < 0.05$). SAA showed higher values in STEMI at t0 and t1. In both groups, TGF-beta had an increase at t1 and t2 with respect to admission, while IL-6 had a decreasing trend.<br>Correlation with cardiac function: A statistically significant correlation was found between SAA levels at admission and cardiac function across all patients:<br>One death was observed in the STEMI group, with SAA levels of 55.3 (7.7–152), compared to three deaths in the NSTEMI group, with SAA levels of 7.2 (5.0–26.9).<br>The study had a prospective cohort design.	A negative correlation with ejection fraction (Kendall Tau = -0.417, $p < 0.05$), indicating that higher SAA levels were associated with worse EF.<br>A positive correlation with WMSI (Kendall Tau = 0.399, $p < 0.05$), indicating that higher SAA levels were associated with worse wall motion.<br>WCW (cell/ul) STEMI 11,850 (9810 - 13,745) vs 8,460 (7,610-11,460)
Ehlers, R. Et al. 2002. USA<br>Correlation between ST-T-Segment Changes with Markers of Hemostasis in Patients with Acute Coronary Syndromes	The study included 85 patients (62 men and 23 women) with a clinical diagnosis of acute coronary syndrome. Patients were recruited within the first 12 hours after the onset of rest angina. The population was divided into two groups for analysis: Patients with electrocardiographic abnormalities (ST-T changes) at admission (n=63). Patients without electrocardiographic abnormalities (n=22).	The intervention was the measurement of Serum Amyloid A levels. Measurements were taken from blood samples at two time points: At admission (0 days). At 48 hours (2 days).	The main comparison of the study was between the SAA levels of the patient group with ST-T changes on the electrocardiogram versus the group without ST-T changes.	The NSTEMI group had SAA levels of 117 ± 69 mg/L compared to 192 ± 83 mg/L in the STEMI group, 2 days after symptom onset.	Values at admission (0 days): Group with ST-T changes: 80 ± 37 mg/L. Group without ST-T changes: 4.3 ± 0.8 mg/L.<br>Values at 48 hours (2 days): Group with ST-T changes: 192 ± 83 mg/L. Group without ST-T changes: 117 ± 60 mg/L.<br>Serum Amyloid A was measured using a rapid laser immunonephelometric assay. The study design was a prospective cohort.<br>It did not calculate or report a Relative Risk (RR) for Serum Amyloid A.	Patients with ST-T-changes had higher fibrinogen and thrombin-antithrombin III complex (TAT) levels than patients without ECG alterations at both times (fibrinogen: 0d: 492 ± 38 vs. 357 ± 36 mg/dl, $p < 0.01$; 2d: 633± 55 vs. 440 ± 50 mg/dl, $p < 0.02$; TAT: 0d: 7.2 ±1.3 vs. 3.6 ±0.7ug/l, $p < 0.05$; 2d: 5.3 ±0.9 vs. 3.2 B 0.5 ug/l, $p < 0.05$).
Harb, T. Et al., 2002. USA<br>Association of C-Reactive Protein and Serum Amyloid<br>A With Recurrent Coronary Events in Stable Patients<br>After Healing of Acute Myocardial Infarction	A total of 1,045 adult patients enrolled in the multicenter, prospective THROMBO study. All participants had sustained a confirmed acute myocardial infarction, diagnosed based on clinical presentation, ECG changes, and elevated CK-MB levels.	Measurement of inflammatory biomarkers, primarily Serum Amyloid A and C-Reactive Protein, performed 2 to 3 months post-AMI.	The cohort was stratified into quartiles based on SAA serum levels. Cardiovascular outcomes were compared between the highest quartile (Q4) and the lower three quartiles (Q1–Q3)	The primary outcome was the recurrence of coronary events, defined as either cardiac death or nonfatal myocardial infarction. Over a mean follow-up of 26 months, 68 cardiovascular events were recorded among the 907 patients with complete SAA data	In univariate analysis, a non-significant trend toward increased risk was noted in the highest quartile of SAA: Hazard Ratio = 1.56, 95% Confidence Interval: 0.81–3.02; $p = 0.19$. In multivariate Model 1, adjusted for age and sex, the HR increased to 1.68 (95% CI: 0.85–3.30; $p = 0.13$). A binary risk analysis comparing Q4 versus Q1–3 also failed to reach statistical significance (HR = 1.19; 95% CI: 0.67–2.09; $p = 0.56$).<br>SAA was measured using an automated immunonephelometric assay with a Behring Nephelometer II analyzer.<br>Prospective cohort study	Events over 2 years: 69 patients with measured CRP levels experienced events (7%). Elevated CRP in Q4 showed a significant association in univariate analysis (HR 2.07; $p = 0.03$).
Haverkate, F. Et. al., 1997. UK.<br>Production of C-reactive protein and risk of coronary events in<br>stable and unstable angina	N = 2,121 outpatients with stable or unstable angina, included in the ECAT (European Concerted Action on Thrombosis and Disabilities) Angina Pectoris Study	Ultrasensitive measurement of inflammatory proteins: C-reactive protein and Serum Amyloid A. Performed at admission and correlated with cardiovascular risk at 2-year follow-up.	Global mean SAA: Geometric mean ≈ 3.67 mg/L. No significant difference between angina types: Unstable: 3.72 mg/L Stable: 3.74 mg/L Atypical: 3.42 mg/L → $p = 0.80$	During the 2-year follow-up, 75 coronary events (non-fatal AMI and sudden death) were recorded. For each 1 standard deviation of SAA, RR 1.14 (0.90–1.44) p=0.27	Global mean SAA: Geometric mean ≈ 3.67 mg/L. There's no significant difference between angina types: Unstable: 3.72 mg/L, Stable: 3.74 mg/L, Atypical: 3.42 mg/L (p=0.80)<br>Multicenter prospective cohort study. SAA was quantified using automated microparticle enzyme immunoassays.	Elevated CRP (>3.6 mg/L) was associated with a relative risk of 1.81–2.25, even after adjusting for factors such as age, ejection fraction, medications, and triglycerides. The final adjusted RR was 1.81 (95% CI: 1.01–2.63).
Jonhson, B. Et. Al. 2004. USA<br>Serum Amyloid A as a Predictor of Coronary Artery Disease and Cardiovascular Outcome in Women The National Heart, Lung, and Blood Institute–Sponsored Women's Ischemia Syndrome Evaluation (WISE)	N = 224 women referred for coronary angiography due to suspected coronary artery disease (CAD). All were part of the WISE (Women's Ischemia Syndrome Evaluation) study. There were no recent events such as acute	Serum Amyloid A measurement at the time of enrollment. SAA analyzed as: Continuous value (log-transformed) Categories by percentile (e.g., SAA ≥10.8 mg/L vs <10.8 mg/L)	Comparison between: Women with elevated SAA (≥10.8 mg/L) vs. Women with low SAA (<10.8 mg/L). SAA levels were also compared in women with obstructive versus non-obstructive coronary artery disease.	Composite of cardiovascular death, myocardial infarction, heart failure, stroke, or need for revascularization. Follow-up: 3 years. Association with Cardiovascular Events Events occurred in 30 women (13%). The risk of events in women with elevated SAA	Association with Coronary Artery Disease Women with angiographically significant CAD had higher SAA levels: CAD present: 12.8±12.2 mg/L vs CAD absent: 9.7±10.0 mg/L p=0.05.	

	myocardial infarction or revascularization.			(≥10.8 mg/L) was: HR = 3.1 (95% CI: 1.4–7.0), p=0.007 After adjusting for factors such as age, classic risk factors, C-reactive protein, and extent of disease, SAA was an independent predictor of major cardiovascular events.	Observational prospective cohort study. SAA was measured by a high-sensitivity latex-enhanced immunonephelometric assay	
Karadag, O. Et. al. 2007.Turkey. Novel cardiovascular risk factors and cardiac event predictors in female inactive systemic lupus erythematosus patients	The study included 25 women with inactive systemic lupus erythematosus (SLE)—defined by a disease activity index score < 4—and 22 healthy female controls. All participants were free of major cardiovascular risk factors such as diabetes, hypertension, hyperlipidemia, smoking, or known coronary artery disease.	Measurement of serum levels of Serum Amyloid A and other novel cardiovascular risk markers (e.g., lipoprotein(a), homocysteine, fibrinogen, and high-sensitivity C-reactive protein) from fasting venous blood samples drawn at baseline. Endothelial function was concurrently assessed via flow-mediated dilation (FMD) of the brachial artery during reactive hyperemia	Comparisons were made between the inactive SLE group and healthy controls to determine differences in SAA levels and endothelial function.	Primary outcome: Evidence of endothelial dysfunction, measured as reduced FMD percentage in SLE patients vs controls. Secondary outcomes: Associations between SAA concentrations and endothelial dysfunction.	SAA was measured with IBL- Hamburg Serum Amyloid A Patients with inactive systemic lupus erythematosus: Median SAA level: 5.2 mg/L Interquartile Range (IQR): 2.8–6.9 mg/L Healthy control group: Median SAA level: 4.3 mg/L IQR: 2.2–6.0 mg/L Statistical comparison between groups: p-value = 0.27 (not statistically significant). Observational cross-sectional study. SAA was quantified using ELISA.	CRP levels, in contrast, were significantly higher in the SLE group (median 1.82 vs 0.68 mg/L, p = 0.04), supporting the presence of low-grade inflammation agsjournals.onlinelibrary.wiley.com +13 ResearchGate +13 ScienceDirect +13 . FMD was significantly lower in SLE patients (7.1% ± 2.1) compared to healthy women (11.4% ± 1.2), p < 0.001, indicating endothelial dysfunction despite absence of traditional risk factors
Katayama, T. Et. al. 2006. Japan. Predictors of Sub-Acute Stent Thrombosis in Acute Myocardial Infarction Patients Following Primary Coronary Stenting With Bare Metal Stent	The study enrolled 433 consecutive patients with confirmed acute myocardial infarction who were admitted within 24 hours of symptom onset and underwent primary percutaneous coronary intervention (PCI)	The main exposure was the plasma concentration of SAA measured 24 hours after AMI	The comparison was made between: Patients who experienced cardiac rupture (CR) (n=11) vs. Patients who did not develop CR (n=422).	The primary outcome was the occurrence of cardiac rupture—defined as free wall rupture or ventricular septal perforation—within 30 days following AMI.	SAA levels were significantly higher in the CR group: 715 ± 502 µg/dL in patients with CR vs. 206 ± 294 µg/dL in those without CR (p < 0.0001) In multivariate logistic regression, SAA concentration above the upper tertile (≥290 µg/dL) was a strong independent predictor of CR: Odds Ratio: 8.8 95% CI: 1.7–25.6 p = 0.01 SAA was quantified using a latex-enhanced immunonephelometric assay. The study design was a prospective observational cohort.	A CR patients had higher peak C-reactive protein at 24h (6.91 vs. 3.32 mg/dL; p = 0.003), though it was not an independent predictor. Other factors such as female gender, diabetes, and anterior infarction were more frequent in the CR group but did not reach statistical significance in multivariate analysis. The follow-up period was 30 days, during which CR occurred in 11 patients (2.5%)
Katayama, T. et. Al. 2005. Japan. Japon Prognostic Value of Serum Amyloid A Protein in Patients With Acute Myocardial Infarction	N=280 consecutive patients with acute myocardial infarction admitted within 10 hours of symptom onset. All patients were successfully reperfused by means of primary percutaneous coronary intervention (PCI).	Plasma concentrations of SAA were measured 24 hours after the onset of AMI. Patients were divided into two groups according to the upper quintile of SAA (325 µg/dL): High SAA group (Group H): ≥325 µg/dL (n=56) Low SAA group (Group L): <325 µg/dL (n=224)	Comparison of patient characteristics, laboratory findings, hemodynamic data, treatment strategy, major complications, and left ventricular function in the chronic phase between Group H and Group L	Major Complications (within the first 6 months): Significantly more complications occurred in Group H than in Group L: Cardiac rupture: 5% in Group H vs 0% in Group L (p = 0.0007) Cardiogenic shock: 45% in Group H vs 9% in Group L (p < 0.0001) Subacute thrombosis (SAT): 11% in Group H vs 1% in Group L (p = 0.0007) Ventricular fibrillation: 23% in Group H vs 6% in Group L (p = 0.0002) Pulmonary edema: 54% in Group H vs 19% in Group L (p < 0.0001). 6-Month Mortality: Nineteen patients died from cardiovascular causes. There were 11 deaths in Group H (16%) vs 7 deaths in Group L (3%) (p = 0.0003).	SAA was identified as an independent predictor of 6-month mortality by multivariate analysis (Risk Ratio: 5.8, 95% CI: 1.3–27.7, p = 0.03). The study design is a prospective observational cohort study. Serum Amyloid A was measured using a high-sensitivity latex-enhanced immunonephelometric assay.	Left Ventricular Function in the Chronic Phase (21 ± 7 days after onset): LV ejection fraction was significantly lower in Group H (52 ± 14%) compared to Group L (57 ± 13%) (p = 0.03).
Mattioli, A. Et. Al. 2005. Italy. Acute myocardial infarction in young patients: nutritional status and biochemical factors	Study group (young MI): 40 male patients with a mean age of 36.7 ± 1.16 years, admitted with acute myocardial infarction. Control group (older MI): 40 male patients with a mean age of 66.3 ± 4.24 years, also admitted with AMI."	Measurement at hospital admission of: C-reactive protein Serum amyloid A protein Serum iron (Fe) Fibrinogen Additional measurements during hospital stay (days 1, 3, 5, and 10 after admission): CRP SAA Lipid levels (total cholesterol, HDL-C, LDL-C, triglycerides) Glucose	Comparison of biochemical factors and nutritional status between the young MI patient group and the older MI patient group at admission. Comparison of the increase in acute phase reactants (CRP and SAA) and dyslipidemic aspects during the hospital stay between the two age groups.	SAA increased significantly in both groups, peaking on day 3 or 5, and levels remained significantly higher in the young group compared to the older group at all time points (p < 0.001 for all days).	SAA: Significantly higher in young patients (101.3 ± 18.2 mg/L) vs. older patients (22.7 ± 15.0 mg/L) (p < 0.001). The study design is a prospective observational cohort study. SAA was measured using a high-sensitivity latex-enhanced immunonephelometric assay.	CRP increased significantly in both groups after MI, reaching a peak on day 3. The CRP level remained significantly higher in the young group compared to the older group at all time points (p < 0.001 for all days). Dyslipidemia, obesity, and hypertension: More prevalent in young patients. Diabetes: More prevalent in older patients
Miyazaki, T. 2014. USA. Plasma Pentraxin 3 Levels Do Not Predict Coronary Events but Reflect Metabolic Disorders in Patients with Coronary Artery Disease in the CARE Trial	749 patients with a prior myocardial infarction that occurred 3 to 20 month before enrollment	Measured plasma PTX3 , SAA levels at baseline by high-sensitivity ELISA in 413 cases with recurrent myocardial infarction or coronary death during a 5-year follow-up period	Measured plasma PTX3 y SAA levels 366 controls	Association of plasma PTX3 levels with recurrent coronary events. 413 patients experienced the primary endpoint of coronary death or MI	PTX3 levels did not correlate with levels of the short pentraxin CRP, and very weakly and positively correlated with another inflammatory marker, SAA Participants with higher PTX3 concentrations also had higher levels of the inflammatory marker SAA, but not of CRP. No significant difference in plasma PTX3 levels occurred between the cases and controls. PTX3 did not predict CHD risk (nonfatal MI and fatal CHD). The SAA levels were measured using a high-sensitivity assay. Prospective nested case-control.	High-sensitivity CRP (hs-CRP) (mg/L): SLE: 4.6 (7.0) Control: 5.5 (7.3) p = 0.06 → Close to significance but not statistically significant SAA (mg/L): SLE: 6.9 (21.4) Control: 7.2 (16.8) p = 0.79 → No significant difference
Morrow. D. Et. Al. 2000. USA	Men and women hospitalized for unstable angina or non–Q-wave	Measurement of Serum Amyloid A levels in serum samples obtained at the	The study conducted comparisons between groups of patients defined	The objective was to predict all-cause mortality at 14 days. The overall mortality rate in	The median SAA concentration was significantly higher in	Mortality by combined risk: Risk stratification showed a clear increase in mortality: 0.7% in the low-risk group,

Serum Amyloid A Predicts Early Mortality in Acute Coronary Syndromes: A TIMI 11A Substudy	myocardial infarction (NQMI). Sample: Of the 630 patients enrolled in the TIMI 11A trial, a total of 435 patients (69%) were included in this substudy, for whom complete serum marker data were available.	time of study enrollment. Concentrations were divided into quintiles, with a focus on the highest quintile (quintile 5) as an indicator of marked elevation. Secondary intervention: A rapid qualitative test for cardiac troponin T (cTnT) was performed. An 'early positive' result was defined as one that appeared in 10 minutes or less, indicating higher concentrations of the marker	by their SAA levels and cTnT results to assess risk. Primary Comparison: Patients in SAA quintile 5 (markedly elevated levels) were compared with patients in SAA quintiles 1–4 (lower levels). Combined Comparison: Patients were stratified into three risk groups to compare their mortality rates: Low risk: Patients with SAA in quintiles 1–4 and a negative rapid cTnT test. Intermediate risk: Patients with SAA in quintile 5 or an early positive result on the rapid cTnT test. High risk: Patients with SAA in quintile 5 and an early positive result on the rapid cTnT test.	the study population was 1.6%. The mortality rate was dramatically higher in the group with the highest SAA levels (quintile 5) compared to the lower SAA groups (5.8% vs. 0.6%, p=0.001). This difference persisted even among patients with a negative troponin T test (6.1% vs. 0.7%)	patients who died compared to those who survived (6.28 mg/dL vs. 0.75 mg/dL, p=0.002). Quantitative determination of serum SAA was performed using a nephelometric immunoassay (specifically, the N Latex SAA assay) on a BN II automated nephelometer. This is a prospective cohort substudy. RR= 9.7 IC 95% 1.97 a 50.68 p = 0.001	3.6% in the intermediate-risk group, and 9.1% in the high-risk group—a statistically significant trend (p<0.002). This association persisted even in the subgroup of patients who had a negative result for troponin T (cTnT), a marker of cardiac injury (mortality of 6.1% vs. 0.7%, p=0.003).
Narins, C. Et. al. 2004. USA. Relationship Between Intermittent Claudication, Inflammation, Thrombosis, and Recurrent Cardiac Events Among Survivors of Myocardial Infarction	1,045 patients who had survived at least two months after a myocardial infarction. Eligible patients were 21 years or older and had been admitted to a coronary care unit with a documented acute myocardial infarction	Presence of intermittent claudication. This condition was identified at the time of study enrollment and was clinically defined as discomfort in the calf, hip, and/or buttocks associated with exertion while walking and relieved by rest.	The comparison group consisted of patients from the same cohort of myocardial infarction survivors who did not have intermittent claudication. Specifically, the study compared: 78 patients with intermittent claudication. 966 patients without intermittent claudication.	The primary outcomes were measured over an average follow-up period of 26 months. Patients with intermittent claudication exhibited a significantly higher proinflammatory and procoagulant state compared to those without claudication.	Serum Amyloid A: Patients with claudication: 0.60 mg/dL Patients without claudication: 0.36 mg/dL The p-value for this difference was < .001. The study is a prospective cohort substudy. Serum Amyloid A levels were measured using high-sensitivity assays.	C-Reactive Protein showed the strongest risk association, with an Odds Ratio of 2.41 (p = .002). Fibrinogen had an Odds Ratio of 2.10 (p = .005). D-dimer had an Odds Ratio of 1.75 (p = .046)
Ogasawara, K. Et. Al. 2000. Japon. A serum amyloid A and LDL complex as a new prognostic marker in stable coronary artery disease	140 consecutive patients with stable coronary artery disease. All patients had at least one coronary stenosis greater than 50% in diameter confirmed by coronary angiography.	Measurement of serum levels of a new marker, the serum amyloid A and LDL complex (SAA/LDL complex), at study baseline. This was measured alongside other traditional inflammatory markers such as C-reactive protein and serum amyloid A alone	Patients who experienced an adverse event during follow-up and those who remained event-free. The study compared baseline levels of inflammatory markers and other risk factors between these two groups to identify predictors of poor prognosis.	The primary outcome was the occurrence of adverse events during an 18-month follow-up period. Clinical events: The defined endpoints were cardiac death, myocardial infarction, stroke, and coronary revascularization. During the study, 21 patients experienced one of these events. Risk association: The key statistical finding was the association between the markers and the events.	The SAA/LDL complex was found to be an independent predictor of events. Values are expressed as mean ± standard deviation (SD). Group without clinical events (n=119): 21.77 ± 7.40 µg/ml Group with clinical events (n=21): 30.60 ± 12.67 µg/ml. Odds Ratio (OR) of 2.32 (95% Confidence Interval: 1.05–4.70). This indicates that for every 10 µg/ml increase in the SAA/LDL complex, the odds of experiencing an adverse event increased by a factor of 2.32. The study was a prospective cohort study. The SAA/LDL complex was measured using a sandwich enzyme-linked immunosorbent assay. This specific method employed a human anti-SAA polyclonal antibody as the primary antibody.	For CRP, Clinical Events Group (n=21): The mean CRP level was 0.27 ± 0.40 mg/dL. No Clinical Events Group (n=119): The mean CRP level was 0.18 ± 0.27 mg/dL.
Olsson, M. Et. al 2011. Expression of the selenoprotein S (SELS) gene in subcutaneous adipose tissue and SELS genotype are associated with metabolic risk factors	The population for the SAA analysis consisted of 90 sibling pairs (180 individuals in total) from the Swedish Obese Subjects (SOS) Sib Pair Study, with each pair including one lean sibling and one sibling with obesity.	The main "intervention" or exposure was the measurement of Serum Amyloid A gene expression in subcutaneous adipose tissue samples. Expression was measured using DNA microarrays.	Comparisons were made as follows: SELS expression levels and metabolic markers were compared between lean siblings and their obese siblings. Metabolic markers were compared among individuals with different SELS gene genotypes (variants). The frequency of SELS genotypes was compared between patients with coronary artery disease (cases) and healthy controls. In the in vitro study, SELS expression in adipocytes was compared before (basal state) and after insulin stimulation.	The quantitative and association result for SAA was as follows: Expression levels: Obese patients showed higher SAA gene expression levels in adipose tissue compared to lean individuals. Quantitative values (microarray signal): Obese group: 448 ± 163 Lean group: 367 ± 130 The study found that the 5227GG genotype of the SELS gene was overrepresented in the group of patients with coronary artery disease (cases). The reported odds ratio for this genotype was 1.28, with a 95% confidence interval of 1.008 to 1.636 (P = .043).	Association with SELS: A key finding of the study was that, although SELS is a putative receptor for SAA, no statistically significant correlation was found between SAA gene expression and SELS gene expression in adipose tissue in either group (lean or obese) after adjusting for age and sex<br><br>Case-control study design. SAA gene expression was measured in subcutaneous adipose tissue. The measurement was performed using DNA microarray analysis (specifically, the Affymetrix Human Genome U133 Plus 2.0 array).	
Ridker, P. Et. Al.2000 C-reactive protein and other markers of inflammation in the prediction of cardiovascular disease in women	The population was part of a nested case-control study within the Women's Health Study, which included apparently healthy postmenopausal women. The population for this analysis consisted of: 122 cases: Women who experienced a cardiovascular event (coronary death, nonfatal myocardial infarction, stroke, or revascularization) during a 3-year follow-up. 244 controls: Women who	The main "intervention" or exposure was the measurement of plasma Serum Amyloid A levels from blood samples collected at baseline.	The study performed two main types of comparisons: Baseline SAA levels were compared between the case group (women with events) and the control group (women without events). The entire population was divided into quartiles based on SAA levels, and the risk of events in the higher quartiles was compared to that of the lowest quartile (reference group).	The quantitative and association results for SAA were as follows: Mean levels: Women who experienced cardiovascular events had significantly higher SAA levels than those who did not. Event group: Mean SAA level = 0.63 mg/dL Non-event group: Mean SAA level = 0.52 mg/dL The difference was statistically significant (P = 0.003). Risk by quartiles (univariate analysis): Cardiovascular event risk	Women in the highest SAA quartile had a 3.0-fold higher relative risk of events compared to those in the lowest quartile (95% Confidence Interval: 1.5 to 6.0) (P = 0.002). The study used a nested case-control design. Plasma levels of Serum Amyloid A were measured using enhanced latex immunonephelometric assays on a BN II analyzer.	Interleukin-6 (IL-6): Levels were significantly higher in women who experienced cardiovascular events (cases) compared to those who did not (controls) (P = 0.003). The relative risk for the highest quartile compared to the lowest was 2.2 (P for trend = 0.02). Soluble Intercellular Adhesion Molecule-1 (sICAM-1): Levels were significantly higher in cases (P = 0.03). The relative risk for the highest quartile was 2.6 (P for trend = 0.004). Homocysteine: Levels were significantly higher in cases (P = 0.02). The relative risk for the highest quartile was 2.0 (P for trend = 0.02).

	remained free of cardiovascular disease, matched to the cases by age and smoking status.			increased with higher SAA levels.		
Samaras, K. Et. A. 2012. Australia. The Value of the Metabolic Syndrome Concept in Elderly Adults: Is It Worth Less Than the Sum of Its Parts?	A total of 930 community-dwelling older adults (aged 70–90 years) were included from the Sydney Memory and Ageing Study.	The intervention was the measurement of Serum Amyloid A levels in fasting blood samples.	The study conducted two main comparisons regarding SAA: Mean SAA levels were compared between participants with metabolic syndrome (MetS) and those without it. The association of SAA levels with the individual components of metabolic syndrome (increased waist circumference, elevated triglycerides, low HDL cholesterol, high glucose, and high blood pressure) was analyzed.	Mean SAA levels: There was no statistically significant difference in mean SAA levels between the groups with and without metabolic syndrome after adjusting for age and sex (P = .13). Without MetS group: 57.8 µg/mL With MetS group: 57.6 µg/mL	No odds ratio or confidence interval was reported for Serum Amyloid A. This was a cross-sectional observational study. Serum Amyloid A was measured using a sandwich enzyme-linked immunosorbent assay.	Association with individual components: Regression analysis found that SAA levels were not associated with the overall diagnosis of metabolic syndrome (P = .22). Instead, SAA levels were independently and significantly associated with three individual components: High waist circumference (P = .001) Low HDL cholesterol (P = .03) High glucose (P = .048)
Wu , T. Et. al. 2007 Establishment of an in-house ELISA and the reference range for serum amyloid A (SAA) Complementarity between SAA and C-reactive protein as markers of inflammation	The study utilized three distinct population groups: Healthy Controls: 201 apparently healthy individuals without risk factors, used to establish the reference range. Diabetes Patients: 31 patients with early-stage type 2 diabetes mellitus (T2DM) without additional clinical complications. Infarction Patients: 38 patients with acute myocardial infarction.	The intervention was the measurement of Serum Amyloid A levels in serum samples. This measurement was performed using a one-step sandwich ELISA assay developed internally by the researchers.	The study performed several key comparisons: SAA levels were compared among the three population groups: healthy controls vs. T2DM patients vs. AMI patients. Within the healthy control group, SAA levels were compared between different age groups (<50 vs. ≥50 years) and between males and females. The positive detection rate (diagnostic sensitivity) of SAA was compared with that of C-reactive protein in the T2DM and MI groups.	SAA levels reflected the degree of inflammation in each condition. Positive Rate (Sensitivity): In T2DM patients, the rate of elevated SAA was 16%. In MI patients, the rate of elevated SAA was 84%.	The primary objective of the study was to develop a measurement assay and compare SAA levels among different groups, not to predict disease risk. Healthy Controls: 2.4 ± 2.1 mg/L T2DM Patients: 5.25 ± 7.9 mg/L (slightly elevated) MI Patients: 242.7 ± 379 mg/L (markedly elevated).<br>The design corresponds to a cross-sectional comparative study. SAA was measured using a sandwich enzyme-linked immunosorbent assay.	Complementarity with CRP: In T2DM, SAA proved to be complementary to CRP. While SAA alone had a positive rate of 16% and CRP 28%, measuring both markers together increased sensitivity to 34%.
Zairis, M. Et. Al. 2007. Greece Association of inflammatory biomarkers and cardiac troponin I with multifocal activation of coronary artery tree in the setting of non-ST-elevation acute myocardial infarction	The study population consisted of 519 consecutive patients with non-ST-segment elevation acute myocardial infarction , who underwent coronary angiography during their hospitalization.	The intervention was the measurement of Serum Amyloid A levels in blood samples taken within the first 12 hours of NSTEMI onset.	The study performed several key comparisons based on angiographic findings: SAA levels were compared among patients grouped by the number of complex coronary lesions (1, 2, or ≥3 lesions). SAA levels were compared between patients with and without a complex lesion containing a visible thrombus. SAA levels were compared between patients with severe coronary artery disease (3-vessel disease or left main trunk disease) and those with less severe disease.	The quantitative and association results for SAA indicated a strong relationship with widespread activation of the coronary tree, but not with disease severity. Association with number of complex lesions: A gradual and significant increase in SAA levels was observed as the number of complex lesions increased (P < 0.001). 1 complex lesion: Mean SAA = 0.4 mg/dL 2 complex lesions: Mean SAA = 2.3 mg/dL ≥3 complex lesions: Mean SAA = 4.3 mg/dL Association with presence of thrombi: Patients with a visible thrombus had significantly higher SAA levels than those without thrombus (mean SAA 2.5 mg/dL vs. 1.1 mg/dL; P < 0.001).	In multivariate analysis, SAA was an independent predictor of having multiple (≥2) complex lesions, with a relative risk (RR) of 1.2 per unit increase in SAA (95% Confidence Interval: 1.1 to 1.3).<br>The design was a prospective cohortstudy. Serum SAA levels were measured using an enzyme-based immunoassay.	Troponin I (cTnI) was an independent and strong predictor of having a lesion with a visible thrombus, with a relative risk (RR) of 3.1 (95% Confidence Interval: 2.5 to 3.8).<br>La PCR fue el predictor independiente más potente de tener múltiples lesiones complejas, con un riesgo relativo (RR) de 4.6 (Intervalo de Confianza del 95%: 2.2 a 9.8). La PCR también fue un predictor independiente muy fuerte de tener una lesión con un trombo visible, con un riesgo relativo (RR) de 8.1 (Intervalo de Confianza del 95%: 4.9 a 13.5).
Zamani, P. Et al. 2012. USA. Inflammatory Biomarkers, Death, and Recurrent Nonfatal Coronary Events After an Acute Coronary Syndrome in the MIRACL Study	The population consisted of 2,923 subjects from the MIRACL (Myocardial Ischemia Reduction with Aggressive Cholesterol Lowering) study who had been hospitalized for an acute coronary syndrome (unstable angina or non–Q-wave myocardial infarction).	The intervention was the measurement of Serum Amyloid A concentration in blood. Samples were collected between 24 and 96 hours after hospital admission for acute coronary syndrome.	The study conducted two types of comparisons: Mean SAA levels were compared among patients who died, those who experienced a recurrent nonfatal coronary event, and those who had no events during follow-up. The population was divided into quartiles based on SAA levels, and the risk of events in the higher quartiles was compared to that of the lowest quartile (reference group).	The results, assessed over a 16-week follow-up period, showed that SAA was selectively associated with the risk of death, but not with recurrent nonfatal events. Association with death: There was a statistically significant association between increasing SAA levels and the risk of death. This association remained significant even after adjusting for other risk factors (p for trend = 0.045). Association with nonfatal events: No significant association was found between SAA levels and the risk of recurrent nonfatal coronary events (p for trend = 0.52)	Median SAA levels: Patients who died had the highest median SAA levels (2.5 mg/dL), compared to those with nonfatal events (1.4 mg/dL) and those without events (1.6 mg/dL).<br>For 16-week mortality risk, SAA showed a significant association. Hazard Ratio : 1.41 95% Confidence Interval: 0.82 to 2.43. For the risk of recurrent nonfatal events (myocardial infarction or unstable angina), SAA showed no association. Hazard Ratio: 0.96 95% Confidence Interval: 0.70 to 1.33 Prospective analysis of a cohort from a clinical trial.	CRP and IL-6 were both significantly associated with an increased risk of death in the initial analysis (p = 0.006 for CRP; p < 0.001 for IL-6). CRP maintained this significant association even after adjusting for other risk factors (p = 0.034). VCAM and tPA were also associated with an increased risk of death in the univariate analysis (p < 0.001 and p = 0.021, respectively).
Zhu, F. et. Al. 2023<br><br>The Netherlands.<br>Plasma Amyloid-b in Relation to Cardiac Function and Risk of Heart Failure in General Population	The study was conducted using data from the "Rotterdam Study," a population-based prospective cohort in the Netherlands. The final population included in the analysis was: Number of participants: 4,156 individuals. Average age: 71.4 years. Sex: 57.1% women (2,373). Key exclusion criteria: At baseline, participants had no established diagnosis of dementia or heart failure.	Amyloid-β, measured in blood samples collected between 2002 and 2005: Aβ40, Aβ42, Aβ42/Aβ40 ratio. For statistical analysis, Aβ40 and Aβ42 levels were log-transformed to approximate a normal distribution.	The study compared the risk and cardiac function markers among individuals with different levels of Aβ biomarkers. The comparison was performed in two ways: Continuous analysis: The effect per 1 SD increase in log-transformed Aβ40 and Aβ42 levels was evaluated. Quartile analysis: Participants were divided into four groups (quartiles) based on their Aβ levels to visualize the cumulative incidence of heart failure.	Over a mean follow-up period of 10.2 years, the cohort recorded 472 new cases of heart failure. The analysis revealed that: A 1-SD increase in Aβ40 was linked to a 32% higher risk of incident HF (Hazard Ratio [HR]: 1.32; 95% CI: 1.15–1.51). This association was significant in men (HR: 1.31; 95% CI: 1.14–1.54), but not in women (HR: 1.06; 95% CI: 0.93–1.22), suggesting a potential sex-specific effect. Higher levels of Aβ42 were initially associated with increased HF risk (HR: 1.12; 95% CI: 1.02–1.24), but this association became nonsignificant after adjusting	Aβ40 value: The mean plasma Aβ40 level among participants was 256.4 pg/mL (interquartile range: 227.9 to 289.0 pg/mL).<br>Association with Heart Failure: A 1 standard deviation increase in log-transformed Aβ40 levels was associated with a 32% higher risk of developing heart failure (HR: 1.32; 95% CI: 1.15–1.51). Aβ42 levels: The mean plasma Aβ42 level among participants was 10.3 pg/mL (interquartile range: 8.8 to 11.9 pg/mL).	Relevance of the Aβ42/Aβ40 Ratio No relevance for cardiac function or heart failure: The Aβ42/Aβ40 ratio showed no significant association with cardiac function measures (such as LVEF or left ventricular mass) nor with the risk of developing heart failure. However, it was significantly associated with all-cause mortality risk: A lower Aβ42/Aβ40 ratio was associated with a higher risk of death from any cause during follow-up (HR: 0.90; 95% CI: 0.85–0.95).

				for Aβ40 (HR: 1.03; 95% CI: 0.92–1.16). The Aβ42/Aβ40 ratio was not associated with HF risk.	Association with Heart Failure:<br>Initially, higher Aβ42 levels were associated with a 12% increased risk of heart failure (HR: 1.12; 95% CI: 1.02–1.24).<br>However, the study clarifies that this association was attenuated and became non-significant after adjusting for Aβ40 levels (HR: 1.03; 95% CI: 0.92–1.16), suggesting that the relationship between Aβ and heart failure is mainly driven by Aβ40 rather than Aβ42.<br>The quantification of Serum Amyloid A was performed using a latex-enhanced immunonephelometric assay. The study design was a prospective cohort.	
Despa, S. Et. al. 2014. USA. Hyperamylinemia Cardioprotection by Controlling Hyperamylinemia in a"Humanized" Diabetic Rat Model	N=20; 5 HF-O, 6 O-NF, 4 H-NF, L-HF<br>N=32, 17 prediabetes hip rats, 15 Age-matched wild-type	Rats= The amylin oligomerization reaction was started in saline solution at 37°C using 50 lmol/L recombinant human amyli	Control: Patients with heart failure without obesity	To determine the role of amylin aggregation in patients with obesity and heart failure and its impact on cardiac myocyte injury.	Amylin. Western blot analysis showed that the level of oligomerized amylin (i.e., trimers, tetrameres and octamers) was significantly higher in O-HF group myocyte lysate compared to control groups L-NF and L-HF.<br>Western blot analysis with an antiamylin antibody on left ventricular myocyte lysates from human hearts demonstrates the presence of amylin trimers (12 kDa), tetramers (16 kDa), and octamers (32 kDa) attached to the sarcolemma or inside the myocytes, comparado con los controles (HF no obesos).<br>Aggregated amylin was even elevated in the O-NF group indicating an early process of amylin aggregation<br>amylin trimers and tetramers are present in both left ventricular protein homogenates and myocyte lysates from the same patient. serial staining with amylin<br>and Congo Red suggested that amylin may form amyloid-like structures in the diabetic human heart. Amylin also accumulates in coronary arteries and cardiac parenchyma in the HIP rat	The results show that non-reduction and non-denaturation conditions preserve large amylin aggregates (> 150 kDa) which can be formed "intrinsically" in cells and tissues.
Hall, L. et. al. 2023. Australia. Amyloid beta 42 alters cardiac metabolism<br>and impairs cardiac function in male mice<br>with obesity	Male C57BL/6J mice fed a high-fat diet for 16 weeks to induce obesity and metabolic dysfunction. Additionally, transgenic APP/PS1 mice overexpressing amyloid-beta peptides were used to model elevated endogenous Aβ production. The study focused on both metabolically healthy and obese animals to explore cardiac effects of $A\beta_{42}$ exposure.	Chronic infusion of $A\beta_{42}$ using osmotic minipumps (200 ng/day for 4 weeks), and neutralization via monoclonal antibody against $A\beta_{42}$. Complementary in vitro experiments included exposure of isolated cardiomyocytes to $A\beta_{42}$ and $A\beta_{40}$, assessing mitochondrial function and glucose uptake.	$A\beta_{42}$-treated vs. $A\beta_{40}$-treated or vehicle-treated animals (PBS). Obese vs. non-obese mice. Obese mice treated with $A\beta_{42}$-neutralizing antibody vs. untreated obese controls.	Cardiac Function: Echocardiography revealed diastolic dysfunction (increased E/e' ratio), reduced left ventricular contractility, increased heart mass, and glucose uptake impairment (via 18F-FDG PET). Mitochondrial Function: $A\beta_{42}$ selectively inhibited mitochondrial complex I, impairing oxidative phosphorylation in cardiomyocytes. Therapeutic Response: $A\beta_{42}$ neutralization completely reversed cardiac dysfunction and restored mitochondrial activity in obese mice.	Chronic $A\beta_{42}$ infusion in non-obese mice increased the E/e' ratio by 35% and decreased fractional shortening by 12%, consistent with significant diastolic impairment. Neutralization of $A\beta_{42}$ in obese mice normalized both E/e' ratio and fractional shortening ($p < 0.05$), demonstrating full restoration of cardiac function.<br>Preclinical experimental design in a murine model. $A\beta_{42}$ was quantified in tissue and plasma using enzyme-linked immunosorbent assays.	$A\beta_{40}$ infusion did not alter cardiac structure or function, emphasizing $A\beta_{42}$-specific pathogenicity. In vitro, $A\beta_{42}$ significantly suppressed mitochondrial complex I activity ($p < 0.01$), reducing both basal and maximal respiration in cardiomyocytes.
Despa, S. Et. Al. 2012. USA. Hyperamylinemia Contributes to Cardiac Dysfunction in<br>Obesity and Diabetes<br>A Study in Humans and Rats	Transgenic HIP mice (n = 10–12 per group), which overexpress human islet amyloid polypeptide, representing a model of chronic hyperamylinemia as seen in obesity and type 2 diabetes. Wild-type littermate controls (n = 10–12), matched by age and sex. In addition, C57BL/6 mice (n = 8) were used for intravenous infusion studies. Isolated ventricular myocytes were used for ex vivo analysis (minimum of 3–5 biological replicates per assay)	The main interventions included: Endogenous overproduction of hIAPP in HIP mice. Chronic intravenous infusion of synthetic hIAPP (50 µg/kg/day for 7 days) in C57BL/6 mice. Direct exposure of cardiomyocytes to hIAPP aggregates (10 µM) for 6–24 hours.	HIP mice vs. wild-type littermates to assess the impact of sustained hyperamylinemia.<br>hIAPP-treated mice vs. rIAPP-treated or vehicle-treated mice to distinguish amylin amyloidogenic effects. Cardiomyocytes exposed to hIAPP vs. non-treated controls or treated with amylin inhibitors.	The primary outcomes included: Global cardiac function measured by echocardiography (ejection fraction, diastolic relaxation velocity). Myocardial hIAPP deposition, assessed by immunohistochemistry and electron microscopy. Mitochondrial dysfunction, oxidative stress (ATP levels, ROS, 4-HNE).	HIP mice demonstrated massive cardiac accumulation of hIAPP: Amyloid deposits were confirmed in cardiomyocytes and perivascular areas using Congo red and anti-hIAPP staining. Electron microscopy visualized fibrillar aggregates (~10–15 nm diameter) consistent with amyloid structure. Translational preclinical experimental design. In vivo study using animal models (HIP transgenic mice). In vitro study in isolated cardiac myocytes. To confirm the presence of hIAPP, specific anti-hIAPP immunohistochemistry,	Cardiac Function (Echocardiography) Ejection fraction: HIP mice: 52.1% ± 4.8% Controls: 64.3% ± 3.9% Mean difference: −12.2%, $p < 0.05$ Diastolic relaxation velocity (E′, tissue Doppler): Reduced by ~35% in HIP mice compared to controls ($p < 0.01$). Mitochondrial and Oxidative Stress Mitochondrial ATP production in hIAPP-exposed cardiomyocytes: Decreased by ~35% compared to untreated cells ($p < 0.01$). ROS levels: 3.2-fold increase in hIAPP-treated cells ($p < 0.001$).

					Congo red staining, and transmission electron microscopy were used.	
Altamirano-Bustamante, N. Et. Al. 2020. México.<br>Protein-conformational diseases in childhood: Naturally-occurring hIAPP amyloid-oligomers and early β-cell damage in obesity and diabetes	Children and adolescents aged 9–17 years, classified into four clinical groups: Patients with obesity (n = 47) Patients with type 1 diabetes mellitus (T1DM, n = 60) Patients with type 2 diabetes mellitus (T2DM, n = 39) Healthy controls (n = 16) Participants were recruited from three pediatric hospitals in Mexico. Comprehensive clinical and biochemical assessments were conducted, including anthropometry, lipid profiles, glucose homeostasis, and biomarkers of β-cell injury.	Quantification and morphological characterization of naturally occurring human islet amyloid polypeptide oligomers—referred to as RIAO (Real hIAPP Amyloid Oligomers)—in serum samples. A novel polyclonal antibody (MEX1), developed specifically to recognize cytotoxic hIAPP oligomers, was used in a validated indirect ELISA. Additional in vitro neurotoxicity assays and Western blotting were used to support the biomarker's relevance.	Comparisons were made across the four groups, with lean, healthy children serving as the primary control group. Within the obesity group, participants were further stratified into clusters based on cardiometabolic risk factors to assess the association between RIAO levels and comorbidity burden.	Prevalence and Quantification of RIAO: Obesity: 3.6 ± 2.0 μg/ml T1DM: 3.4 ± 1.5 μg/ml T2DM: 2.8 ± 1.7 μg/ml Controls: 1.6 ± 0.3 μg/ml Significant differences were observed between all disease groups and the control group ($p < 0.001$)	A RIAO level ≥ 3.35 μg/ml effectively identifies children with obesity at elevated risk for β-cell dysfunction and associated cardiometabolic complications.<br>The quantitative performance of this biomarker Area Under the Curve (AUC): 0.83, Sensitivity: 79%, Specificity: 82%) supports its potential clinical utility in early screening strategies.<br>Multicenter, cross-sectional, analytical, ambispective, and blinded study. hIAPP was quantified using an indirect ELISA.	β-Cell Dysfunction and Its Association with RIAO Population: Obese children (n = 47) vs. healthy controls (n = 16) Measured Variables: Glucose, HbA1c, C-peptide, HOMA-IR, triglycerides, HDL, ALT, uric acid, etc. Linear Associations (Simple Regression): HbA1c and RIAO: p = 0.027 → Positive association C-peptide and RIAO: p = 0.017 → Positive association HOMA-IR and RIAO: p = 0.012 → Positive association. Number of Comorbidities and RIAO: Children with ≥4 comorbidities had significantly higher RIAO levels: Median RIAO: 4.52 μg/ml (IQR: 2.23) Children with ≤2 comorbidities: Median RIAO: 1.62 μg/ml (IQR: 1.49) Statistical Test: Welch's test, p = 0.003 Similar results for ≥3 comorbidities ($p < 0.001$)
Jousilathi, P. Et. Al. 2001. Finland. The association of c-reactive protein, serum amyloid a and fibrinogen with prevalent coronary heart disease — baseline findings of the PAIS project	The study included 1,400 Finnish men aged 45 to 74 years, selected from a nationwide cardiovascular risk factor survey conducted in 1997.	The primary exposure was the quantitative measurement of inflammatory biomarkers: C-reactive protein Serum amyloid A Plasma fibrinogen	Participants were stratified into quartiles based on biomarker concentrations, using the lowest quartile as the reference group. This allowed for assessment of a dose–response relationship between biomarker levels and CHD risk.	The primary outcome was prevalent coronary heart disease, verified through national insurance registers and physician-confirmed diagnoses. Associations were analyzed using multivariable logistic regression, adjusting for age, blood pressure, smoking, serum cholesterol, waist-to-hip ratio (WHR), and use of lipid-lowering therapy.	Mean SAA levels were significantly higher in men with CHD: CHD (−): 10.30 ± 44.32 μg/mL CHD (+): 16.48 ± 47.98 μg/mL, $p < 0.001$ Adjusted odds ratios (OR) for CHD by increasing SAA quartile: 2nd quartile: OR 1.68 (95% CI: 1.01–2.79) 3rd quartile: OR 1.83 (95% CI: 1.11–3.03) 4th quartile: OR 2.41 (95% CI: 1.49–3.91), p for trend < 0.001. After further adjustment for WHR (a marker of visceral adiposity), the association remained significant, though somewhat attenuated, indicating that central obesity<br>Cross-sectional study. SAA was quantified using ELISA.	Mean CRP levels were significantly higher among men with prevalent coronary heart disease, measuring 2.98 mg/L (± 4.74) in those with CHD versus 2.06 mg/L (± 3.76) in those without, with a statistically significant difference (p = 0.001). compared to participants in the lowest quartile, those in the third quartile had a 1.67-fold increased risk of CHD (95% CI: 1.01 to 2.76), and those in the highest quartile had a 2.29-fold increased risk (95% CI: 1.39 to 3.77). This association demonstrated a highly significant linear trend (p for trend < 0.0
Meakin, P. Et. Al. 2000. Elevated circulating amyloid concentrations in obesity and diabetes promote vascular dysfunction	The study involved three groups of adult human participants: Lean controls (n = 23) Individuals with obesity but without type 2 diabetes ((n = 22) Individuals with obesity and T2D (n = 22) In addition, the authors employed mouse models and cultured human endothelial cells to investigate mechanistic pathways linking amyloid proteins with vascular dysfunction	The main exposures included measurement and experimental administration of: Serum amyloid A These proteins were quantified using ELISA. Functional assays assessed: Nitric oxide-dependent vasodilation Oxidative stress (ROS production) Mitochondrial dysfunction	Comparisons were made between: Lean vs. obese vs. obese + T2D individuals Endothelial cells treated with hIAPP/SAA vs. vehicle controls Mice infused with hIAPP/SAA vs. controls receiving saline This allowed isolation of the specific effects of SAA and hIAPP on vascular physiology.	Primary outcomes included: Circulating SAA concentrations Degree of endothelial dysfunction (measured by NO bioavailability and vascular relaxation) Inflammatory activation (VCAM-1, ICAM-1, NF-κB pathway) Oxidative and mitochondrial stress	SAA levels were significantly elevated in metabolic disease: Circulating SAA levels (pg/mL) Lean controls: 4.2 ± 0.7 Obese (non-diabetic): 12.6 ± 2.1 (3× higher, $p < 0.01$ vs. lean) Obese + T2D: 24.5 ± 4.9 (~6× higher, $p < 0.001$ vs. lean) Strength of association: There was a strong positive correlation between SAA concentration and metabolic status severity ($r > 0.85$), with T2D patients exhibiting the highest SAA burden, aligning with the degree of vascular impairment.<br>The human component is a cross-sectional study. An in vivo experimental study was conducted using a murine model. SAA was quantified using ELISA.	Vascular Function (Mice infused with SAA, 5 μg/day for 7 days) Endothelium-dependent vasodilation (response to acetylcholine) was reduced by ~45% in SAA-treated mice compared to saline controls ($p < 0.01$). NO production dropped by ~50% in aortic rings isolated from SAA-infused mice ($p < 0.01$). ROS production increased by 3.2-fold ($p < 0.01$) VCAM-1 and ICAM-1 mRNA expression increased 2.5–3.0-fold ($p < 0.001$) NF-κB nuclear translocation rose by 65% vs. untreated cells ($p < 0.01$)
