# Supplementary material for: Circulating Amyloid and Misfolded Biomarkers in Early Myocardial Injury: A Systematic Review and Epistemic Meta-Analysis
Source: Int J Mol Sci. 2026 Sep 7;27(17):7956. doi: 10.3390/ijms27177956 (PMC13566443; doi:10.3390/ijms27177956)
Supplement: Supplementary file 1 [file ijms-27-07956-s001.zip › Table S2. Study Characteristics.pdf]

Title	Research question	Outcome	Methodology	Result according to question	Relevant to question	TOTAL	Type of research
Aleksova,A. 2025	20	20	20	20	20	100	
Aleksova,A. 2025							Revision
Alexander, R. 1994							Revision
Altay, S. 2017.	20	20	10	10	0	60	
Barbati, E. 2012	20	10	10	0	0	40	
Biasucci, L. 2000	20	20	10	10	10	70	
Biasucci, L. 2000							Revision
Biasucci, L. 2004	20	0	20	0	0	40	
Bouzidi, N. 2020.	20	10	10	10	10	60	
Butt, N. 2020	20	10	20	10	0	60	
Cai, X. 2020	20	10	10	0	0	30	
Calderón-Hernández, M. 2021							Revision
Carty, C. 2009	20	10	10	10	10	60	
Casl, M. 1995	20	20	20	20	10	90	
Cederstrom, S. 2024	20	10	20	10	20	80	
Chami, B. 2015	20	10	10	0	0	40	
Chami, B. 2019	20	20	10	0	0	50	
Danesh, J. 2019	20	10	10	10	10	60	
De Backer, J. 2002	10	10	20	0	0	40	
Del Moral, L. 2024	20	20	20	20	20	100	
Despa, S. 2012	20	20	20	20	20	100	
Di Stefano, R. 2009	10	10	20	20	20	80	
Ehlers, R. 2002	20	20	10	20	20	90	
Erdenen, F. 2018	20	20	0	0	0	40	
Eren, E. 2014	10	10	10	20	20	70	
Filep, J. 2008							Revision
Gagno, G. 2020							Revision
Greco, S. 2017	20	10	20	10	10	70	
Hall, L. 2024	20	20	20	10	10	80	
Harb, T. 2002	20	10	20	20	10	80	
Harb, T. 2003	10	0	10	10	0	30	
Haverkate, F. 1997	20	20	20	20	10	90	
Hirschfield, G. 2003							Revision
Hjort, M. 2021	20	10	10	10	20	70	
Hua, S. 2009	10	10	10	10	0	40	
Jang, S. 2023	20	10	10	10	10	60	
Jenny, N. 2007	20	10	10	10	10	70	
Ji, H. 2024							
Johnson, B. 2004	20	20	20	20	20	100	
Jovanovic, D. 2004							Otro idioma
Karadag, O. 2007	20	20	20	10	10	80	

Katayama, T. 2008	20	20	10	0	0	50	
Katayama, T. 2006 Predictors of Sub-Acute	20	20	20	20	20	100	
Katayama, T. 2006 CR	20	10	10	10	10	60	
Katayama, T. 2005	20	20	20	20	10	90	
Khan, F. 2023							Revision
Kiernan, U. 2003	10	20	20	0	0	50	
Korita, I. 2013.	10	10	20	10	0	50	
Liu, M. 2021	20	10	10	10	10	60	
Liuzzo, G. 1999	20	10	10	0	0	40	
Liuzzo, G. 1994	20	10	10	10	10	60	
Lubrano, V. 2015							Revision
Maier, W. 2005	20	10	10	10	10	60	
Mangge, H. 2016							Revision
Matsubara, T. 2003.	20	10	10	10	10	60	
Mattioli, A. 2005	20	20	20	10	20	90	
Miyazaki, T. 2014	20	20	20	10	10	80	
Morrow, D. 2000	20	20	20	20	20	100	
Narins, C. 2004	20	20	20	20	10	90	
Odeberg, J. 2016	20	10	10	10	10	60	
Odeberg, J. 2025	20	10	10	10	10	60	
Ogasawara, K. 2004	20	20	20	10	10	80	
Olsson, M. 2011	20	20	20	10	10	80	
Ridker, P. 2000	20	20	20	10	20	90	
Rodriguez Camargo, D. 2018							Revision
Samaras, K. 2012	20	20	20	10	10	80	
Schillinger, M. 2004	20	10	10	0	0	40	
Shirani, J. 1990							Revision
Stakos, D. 2020							Revision
Stamatelopoulos, K. 2015	20	10	10	10	10	60	
Stefanadis, C. 2000	20	10	10	10	0	50	
Vallejo, A. 2018	20	20	20	10	0	70	
Wang, M. 2017	10	20	10	0	0	40	
Whicher, J. 1999							Revision
Willerson, J. 2002							Revision
Witting, P. 2011	20	20	20	10	0	70	
Wu, T. 2007	20	20	20	10	10	80	
Xu, Y. 2025	20	10	10	10	10	60	
Yeh, K. 2022	20	10	10	10	10	60	
Younger, S. 2020	20	10	10	0	0	40	
Zairis, M. 2007	20	10	20	20	10	80	
Zairis, M. 2005	20	20	20	10	0	70	
Zamani, P. 2013	20	20	20	20	20	100	
Zhu, F. 2023	20	20	20	20	20	100	
Manual							
Altamirano-Bustamante 2020	20	20	20	20	10	90	

Fernandez-Gomez 2018	0	0	0	0	0	0	
Avila Vazquez 2018	0	0	0	0	0	0	
Thomson, S. 1995	20	20	0	0	20	60	
Mueller C, 2002	20	20	0	10	10	60	
Jousilahti P, 2001	20	20	20	10	10	80	
Malle, E. 1996	20	20	0	20	10	70	
Meakin, P. 2020	20	20	20	20	10	90	
Despa, S. Et. al 2014	20	20	20	10	20	90	
