# Supplementary material for: Circulating Amyloid and Misfolded Biomarkers in Early Myocardial Injury: A Systematic Review and Epistemic Meta-Analysis
Source: Int J Mol Sci. 2026 Sep 7;27(17):7956. doi: 10.3390/ijms27177956 (PMC13566443; doi:10.3390/ijms27177956)
Supplement: Supplementary file 1 [file ijms-27-07956-s001.zip › Tables S3 Bias.pdf]

Table S3A. Adapted Newcastle-Ottawa Scale (NOS-xs) for assessing cross-sectional association studies.

Study	Selection		Exposure and Outcome		Confounding factors		Assessment		
**Author, year**	**Q1**	**Q2**	**Q3**	**Q4**	**Q5**	**Q6**	**Total score**	**Quality**	**Risk of bias**
Del Moral LE et al., 2024	1	0	1	2	1	1	6	Regular	Moderate
Altamirano-Bustamante NF et al., 2020	0	0	2	1	0	0	3	Low	High
Samaras K et al., 2012	1	0	1	1	2	1	6	Regular	Moderate
Karadag O et al., 2007	0	0	2	1	0	0	3	Low	High
Jousilahti P et al., 2001	1	0	1	2	2	1	7	Good	Low

Table S3B. Newcastle-Ottawa Scale (NOS) for assessing case-control studies.

Study	Selection				Comparability	Exposure			Assessment		
**Author, year**	**Q1**	**Q2**	**Q3**	**Q4**	**Q1**	**Q1**	**Q2**	**Q3**	**Total score**	**Quality**	**Risk of bias**
Miyazaki T et al., 2014	0	1	1	1	2	1	1	0	7	Good	Low
Olsson M et al., 2011	0	0	1	1	2	1	1	0	6	Regular	Moderate
Ridker PM et al., 2000	1	1	1	1	2	1	1	0	8	Good	Low

Table S3C. Newcastle-Ottawa Scale (NOS) for assessing cohort studies.

Study	Selection				Comparability	Outcome			Assessment		
**Author, year**	**Q1**	**Q2**	**Q3**	**Q4**	**Q1**	**Q1**	**Q2**	**Q3**	**Total score**	**Quality**	**Risk of bias**
Aleksova A et al., 2025	1	1	1	1	2	1	1	1	9	Good	Low
Cederström S et al., 2024	1	1	1	1	2	1	1	1	9	Good	Low
Zhu F et al., 2023	1	1	1	1	2	1	1	1	9	Good	Low
Zamani P et al., 2012	1	1	1	1	2	1	1	1	9	Good	Low
Di Stefano R et al., 2009	1	1	1	1	1	1	1	1	8	Good	Low
Zairis MN et al., 2007	1	1	1	1	2	1	1	1	9	Good	Low
Katayama T et al., 2006	1	1	1	1	2	1	1	1	9	Good	Low
Katayama T et al., 2005	1	1	1	1	2	1	1	1	9	Good	Low
Mattioli AV et al., 2005	0	0	1	0	0	1	0	1	3	Low	High
Johnson BD et al., 2004	1	1	1	1	2	1	1	1	9	Good	Low
Narins CR et al., 2004	1	1	1	1	2	1	1	1	9	Good	Low
Ogasawara K et al., 2004	1	1	1	1	2	1	1	1	9	Good	Low
Ehlers R et al., 2002	0	1	1	0	0	1	1	0	4	Low	High
Harb TS et al., 2002	1	1	1	1	2	1	1	1	9	Good	Low
Morrow DA et al., 1998	1	1	1	1	0	1	1	1	7	Regular	Moderate
Haverkate F et al., 1997	1	1	1	1	2	1	1	1	9	Good	Low
Casl MT et al., 1995	0	1	1	1	0	1	1	1	6	Regular	Moderate

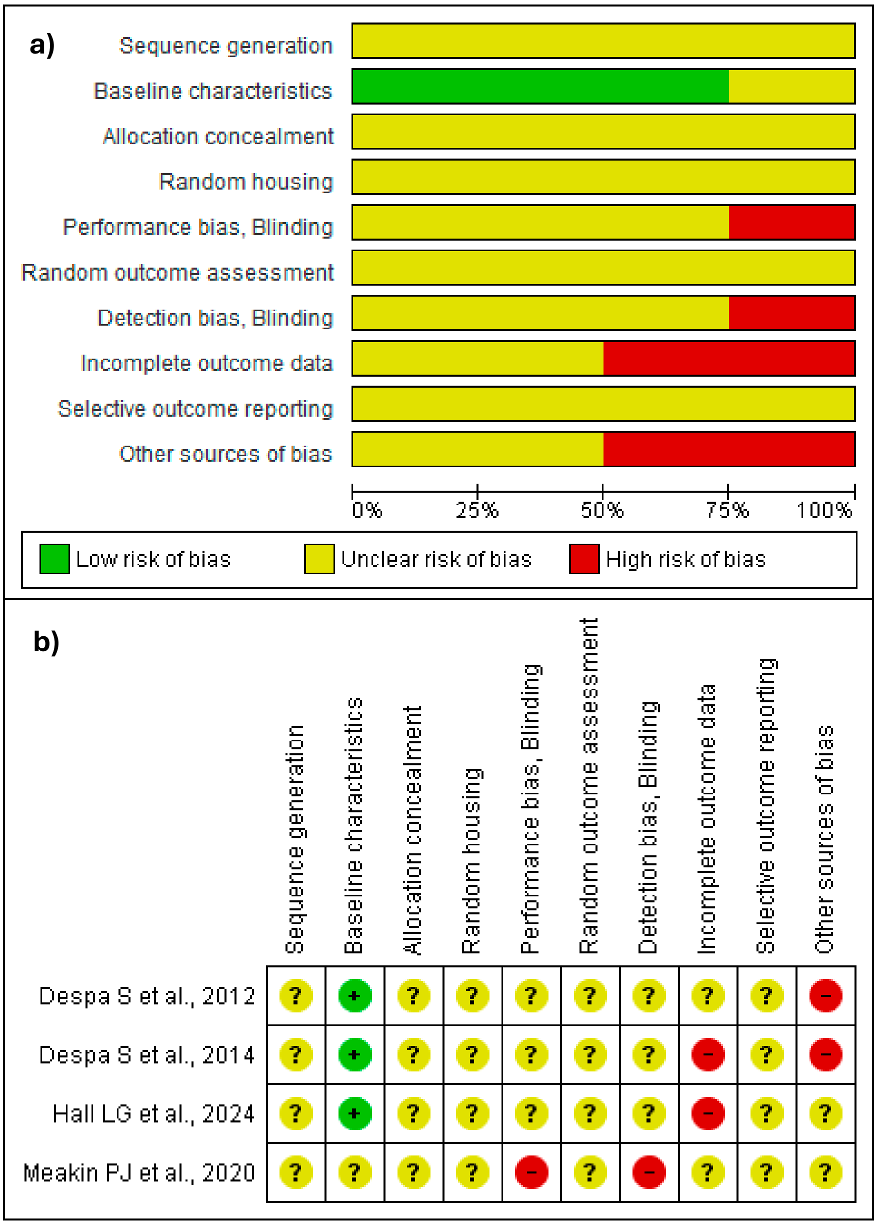

Figure S1. Risk of bias in animal model studies, a) Graph, b) Summary
